# Supplementary material for: Selective targeting of the TLR2/MyD88/NF-κB pathway reduces α-synuclein spreading in vitro and in vivo
Source: Nat Commun. 2021 Sep 10;12:5382. doi: 10.1038/s41467-021-25767-1 (PMC8433339; doi:10.1038/s41467-021-25767-1)
Supplement: Supplementary file 1 — Supplementary Information [file 41467_2021_25767_MOESM1_ESM.pdf]

Selective targeting of the TLR2/MyD88/NF-κB pathway reduces α-synuclein spreading in vitro and in vivo

Debashis Dutta[1,*], Malabendu Jana[1,*], Moumita Majumder[1], Susanta Mondal[1], Avik Roy[1], and Kalipada Pahan[1,2]

[1]Department of Neurological Sciences, Rush University Medical Center, Chicago, USA;
[2]Division of Research and Development, Jesse Brown Veterans Affairs Medical Center, Chicago, USA

[*]First two authors have equal contribution to the work.

Address correspondence to:
Kalipada Pahan, Ph.D.
Department of Neurological Sciences
Rush University Medical Center
1735 West Harrison St, Suite Cohn 310
Chicago, IL 60612
Tel: (312) 563-3592
Fax: (312) 563-3571
Email: Kalipada_Pahan@rush.edu

Table S1: Antibodies used for the study.

Protein	Source	Catalogue No.	Application/ Dilution or amount	Host Species
α-Synuclein	Abcam	ab138501 (MJFR1)	IFC/1:500	Rabbit
α-Synuclein	BD Bioscience	610787	WB/1:1000	Mouse
α-Synuclein	Abcam	ab27766 (LB509)	IFC/1:5000	Mouse
Phospho Ser129 α-synuclein	Abcam	ab51253 (EP1536Y)	WB/1:1000<br>IFC/1:2000<br>IHC/1:2000	Rabbit
Tyrosine hydroxylase (TH)	Pel-Freeze Biologicals	P40101	WB/1:2000<br>IFC/1:1000<br>IHC/1:1000	Rabbit
TH	Immunostar	22941	IFC/1:500	Mouse
Inducible nitric oxide synthase (iNOS)	BD Bioscience	610329	WB/1:1000<br>IFC/1:200	Mouse
Ionized calcium binding adaptor molecule 1 (Iba1)	Abcam	ab5076	WB/1:1000<br>IFC/1:1000<br>IHC/1:1000	Goat
Glial fibrillary acidic protein (GFAP)	Santa Cruz	Sc-6171	WB/1:1000	Goat
GFAP	Abcam	ab53554	IFC/1:1000	Goat
Interleukin-1β (IL-1β)	Santa Cruz	sc-7884	WB/1:1000	Rabbit
Toll like receptor 2 (TLR2)	Abcam	ab16894	WB/1:500	Abcam
MyD88	Santa Cruz	sc-74532	WB/1:1000	Mouse
Acetylated p65	Abcam	ab19870	IFC/1:500	Rabbit
Phospho Ser536 p65	Cell Signaling	3033S	IFC/1:250	Rabbit

P2RY12	Biolegend	848002	IFC/1:100	Rat
Cleaved caspase 3	R&D	MAB835	IFC/1:200	Rabbit
Actin	Abcam	ab8226	WB/1:10000	Mouse
NF-κB p65	Santa Cruz Biotechnology	sc-8008	ChIP/2 µg per reaction	Mouse
NF-κB p50	Santa Cruz Biotechnology	sc-8414	ChIP/2 µg per reaction	Mouse
CBP	Santa Cruz Biotechnology	sc-365387	ChIP/2 µg per reaction	Mouse
p300	Santa Cruz Biotechnology	sc-48343	ChIP/2 µg per reaction	Mouse
RNA polymerase II	Santa Cruz Biotechnology	sc-56767	ChIP/2 µg per reaction	Mouse

WB, Western blot; IFC, Immunofluorescence; IHC, Immunohistochemistry; ChIP, chromatin immunoprecipitation.

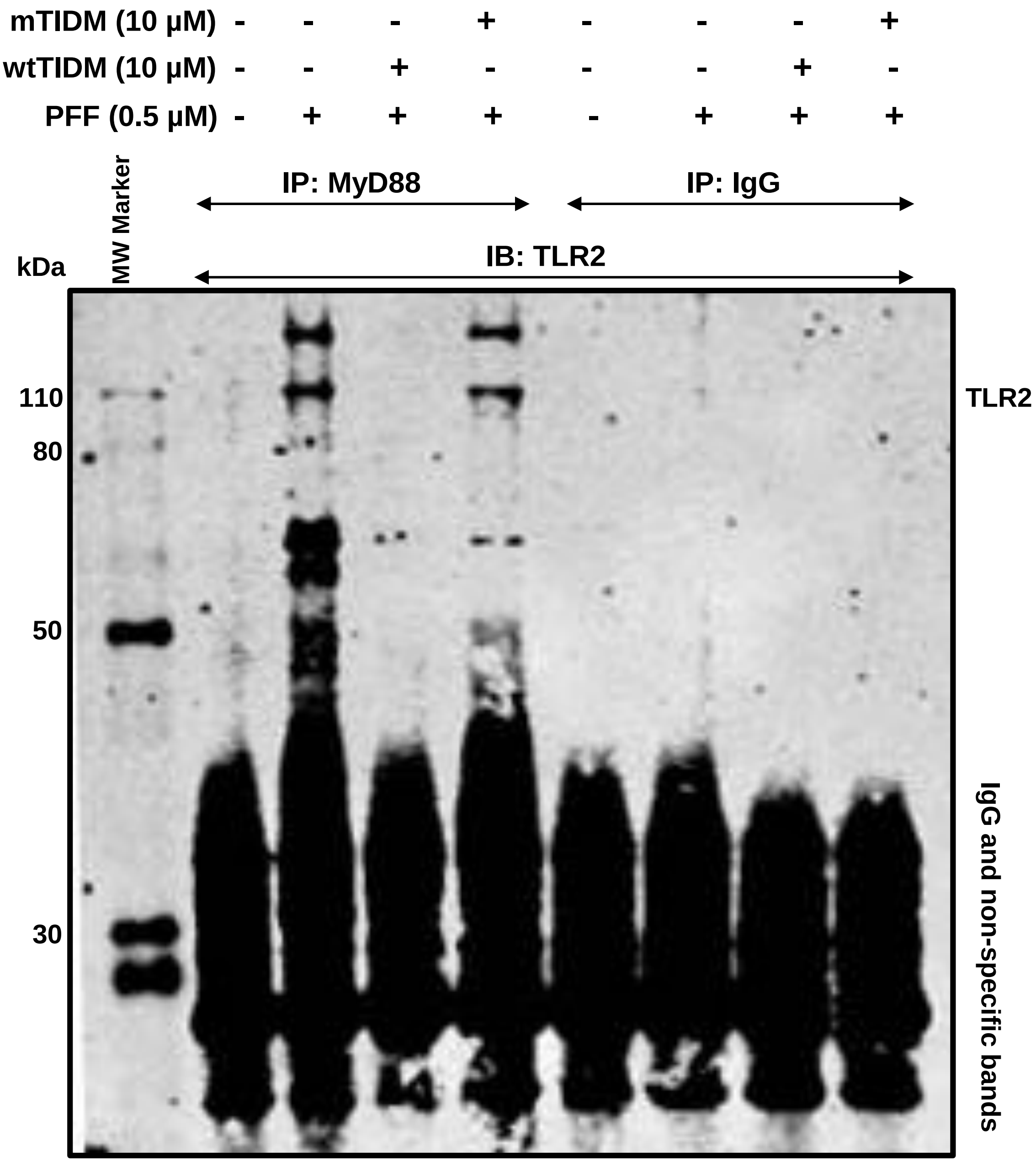

Figure S1. Effect of TIDM on PFF-induced interaction between TLR2 and MyD88 in BV-2 microglial cells. BV-2 cells were pre-incubated with wtTIDM or mTIDM for 1 h were stimulated with α-syn PFF (0.5 µM or 7 µg/ml) under serum-free condition. After 1 h of stimulation, cellular extracts were immunoprecipitated (IP) with an anti-MyD88 antibody, followed by Western blotting of immunoprecipitates for TLR2. As a control, cellular extracts were also immunoprecipitated with normal IgG. All samples were run in a same gel. Results represent three independent analysis (n=3).

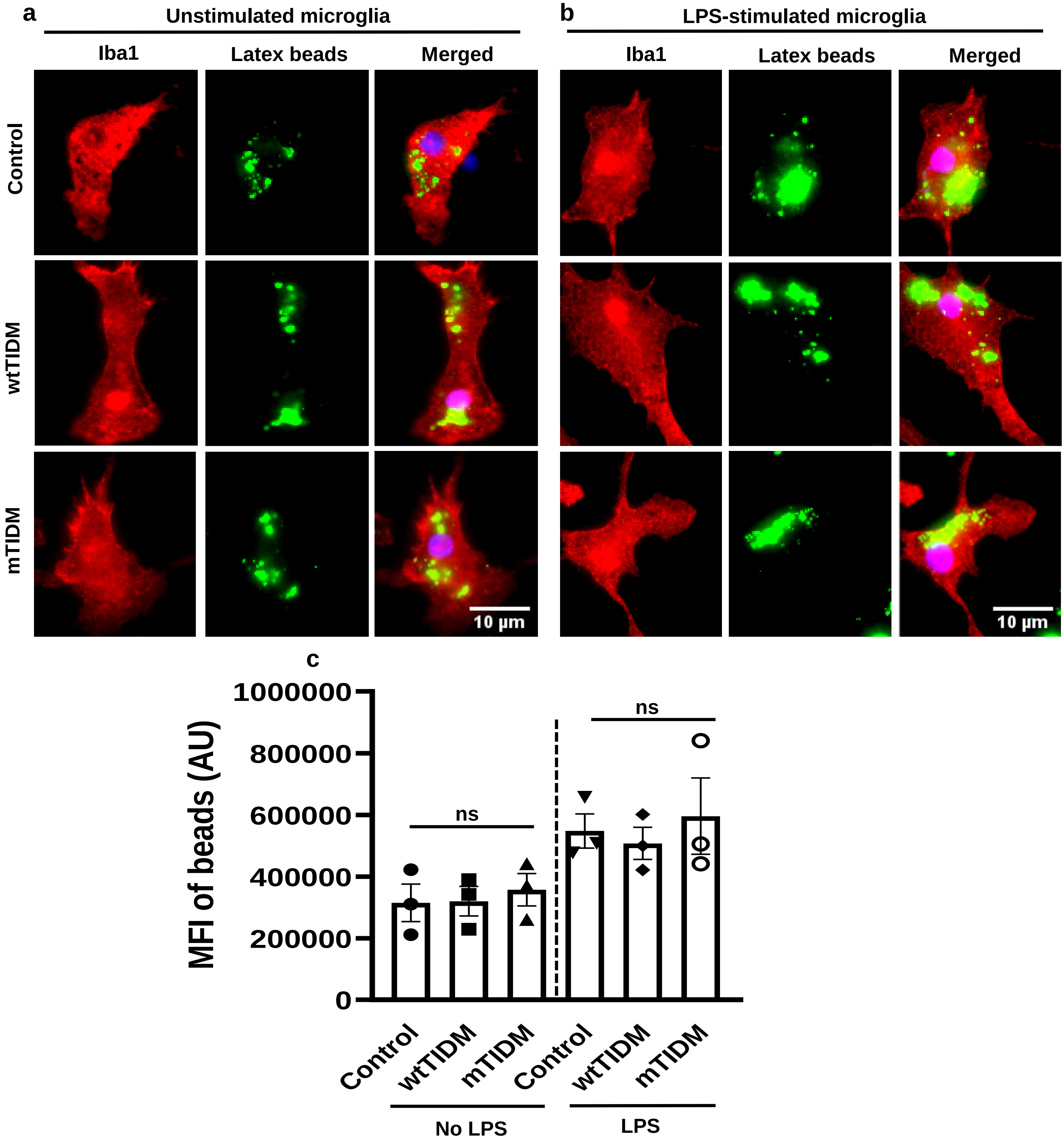

Figure S2. Unaltered microglial phagocytosis by TIDM. Primary microglia were treated with either wtTIDM or mTIDM (5 µM) and after 30 min FITC-tagged latex beads were added to the medium and kept for 2 h. Then immunocytochemistry was performed using Iba1 antibodies and images of cells were captured under fluorescent microscope. The mean fluorescence intensity (MFI) of FITC-tagged beads in microglia was measured by ImageJ (a, c). Similarly the experiment was performed for LPS-stimulated microglia, where following 30 min of TIDM treatment, the cells were stimulated with LPS for 1 h and then the phagocytosis assay was carried out (b, c). Statistical analyses were performed by one-way ANOVA followed by Tukey's multiple comparison tests. Values are given as mean ± SD, 'ns' means non-significant (n=3 different experiments).

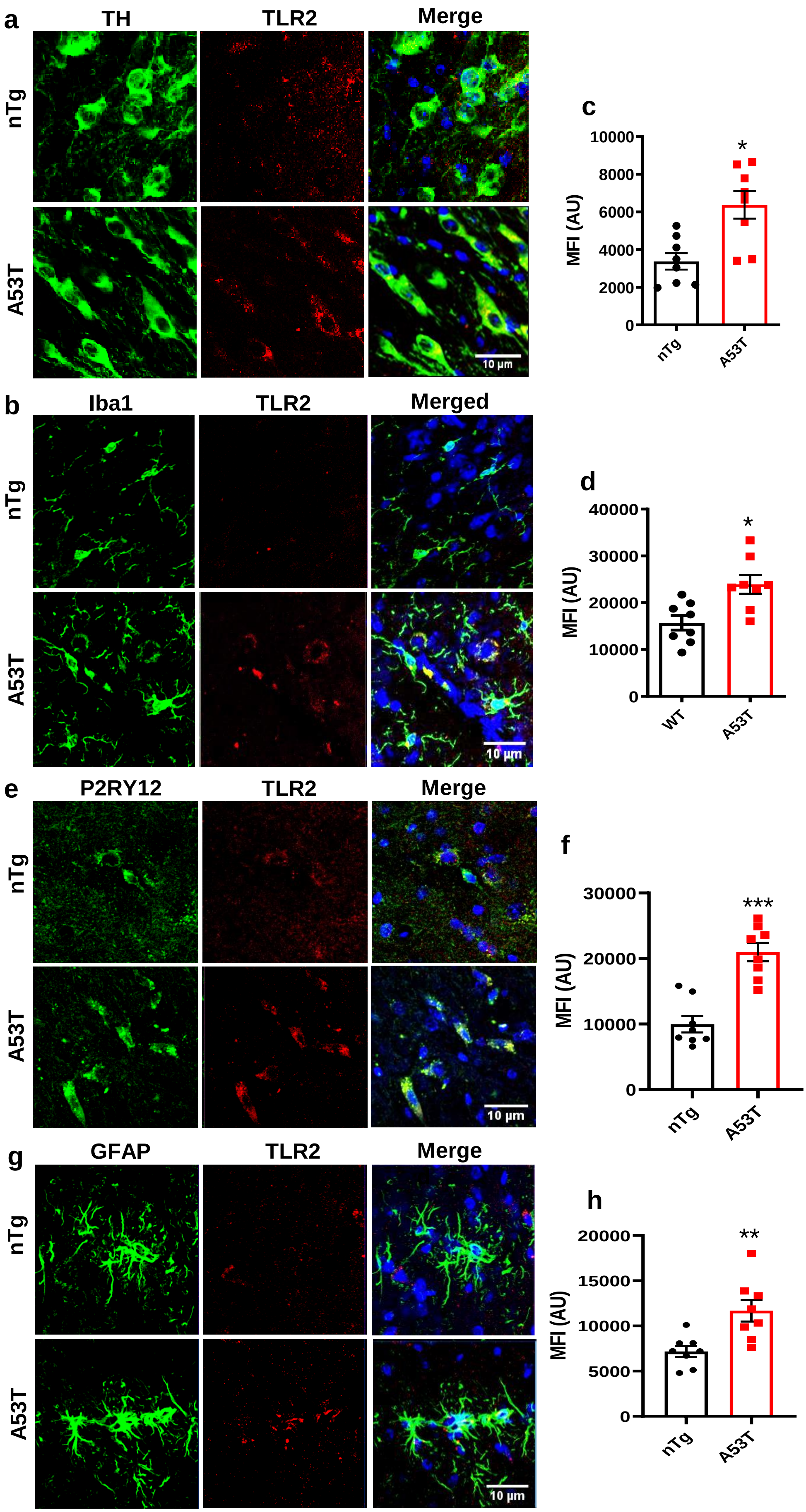

Figure S3. Up-regulation of TLR2 in brain of aged A53T mice. Immunofluorescence images show TLR2 expression (red) in TH neurons (green), in Iba1-positive microglia (green), in P2RY12-positive microglia and in GFAP-positive astrocytes present in substantia nigra (SN) of nTg and 9 months old A53T mice. Images were captured at 60x magnification and the scale bar was 10 µm (a, b, e, and g). MFI of TLR2 expression in both TH neurons (c, p=0.0324), in microglia (d, p=0.0214 and f, p=0.000041) and in astrocytes (h, p=0.0045) of nTg and A53T animals was measured by ImageJ. Statistical analyses were performed by Student's two-tailed unpaired 't-test'. Values are given as mean ± SEM, *p< 0.05, **p< 0.01 and ***p< 0.001 indicate significance compared to nTg (n=4 animals per group).

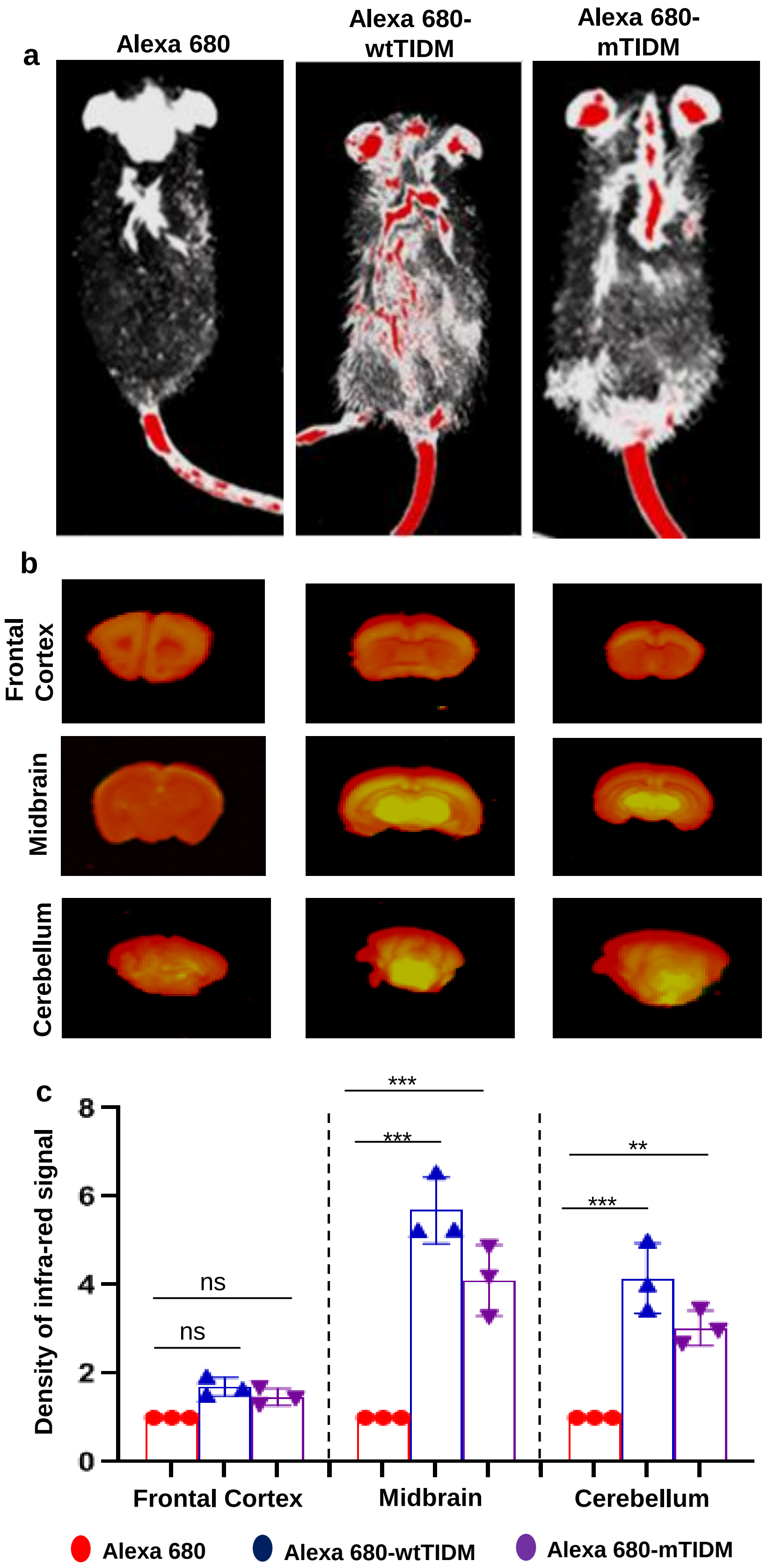

Figure S4. The wtTIDM or mTIDM peptides reach the brain in mice. The bioavailability of TIDM peptides in brain was monitored by injecting animals with Alexa 680 tagged wtTIDM or mTIDM peptides through tail vein and following 2 h of injection, the animals were anesthetized and scanned under the LICOR infrared scanner (a). Presence of TIDM peptides in specific brain regions was monitored by cutting blocks from frontal cortex, midbrain and cerebellum of the mice brains followed by scanning under the infrared scanner (b). Density of infrared signals from different brain regions was measured by ImageJ (c). Student's unpaired 't-test' was performed to determine statistical significance. $^{**}p < 0.01$; $^{***}p < 0.001$; 'ns' means non-significant. For frontal cortex, $p = 0.3418$ and $p = 0.3413$ for Alexa 680 vs Alexa 680+wtTIDM and Alexa 680 vs Alexa 680+mTIDM, respectively. For midbrain, $p = 0.0002$ and $p = 0.0004$ for Alexa 680 vs Alexa 680+wtTIDM and Alexa 680 vs Alexa 680+mTIDM, respectively. For cerebellum, $p = 0.001$ and $p = 0.008$ for Alexa 680 vs Alexa 680+wtTIDM and Alexa 680 vs Alexa 680+mTIDM, respectively. Values are given as mean ± SD (n = 3).

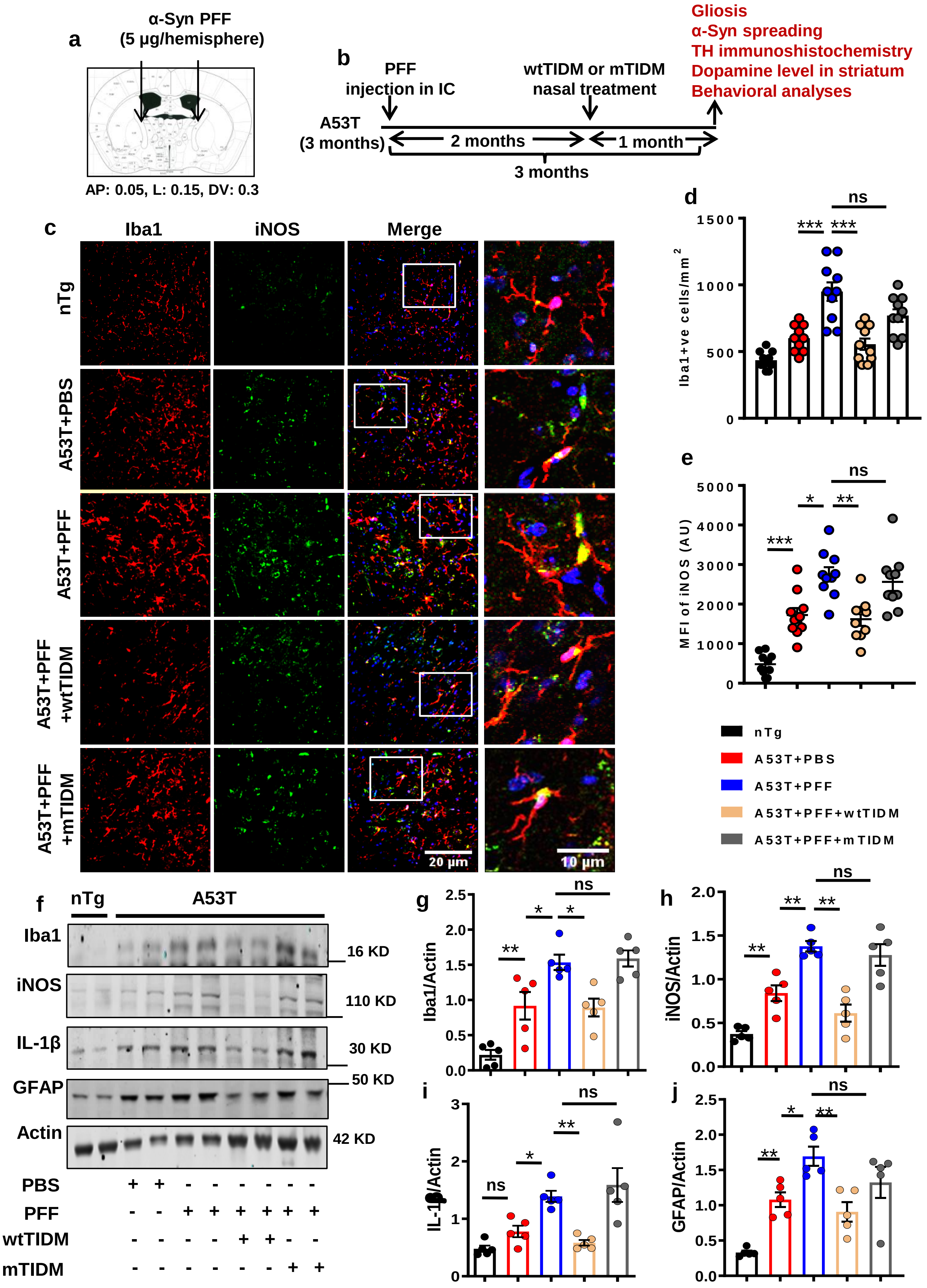
a
α-Syn PFF
(5 µg/hemisphere)
AP: 0.05, L: 0.15, DV: 0.3
b
PFF
injection in IC
wtTIDM or mTIDM
nasal treatment
A53T
(3 months)
2 months
1 month
3 months
Gliosis
α-Syn spreading
TH immunoshistochemistry
Dopamine level in striatum
Behavioral analyses
c
Iba1
iNOS
Merge
nTg
A53T+PBS
A53T+PFF
A53T+PFF +wtTIDM
A53T+PFF +mTIDM
20 µm
10 µm
d
Iba1+ve cells/mm²
e
MFI of iNOS (AU)
nTg
A53T+PBS
A53T+PFF
A53T+PFF+wtTIDM
A53T+PFF+mTIDM
f
nTg
A53T
Iba1
16 KD
iNOS
110 KD
IL-1β
30 KD
GFAP
50 KD
Actin
42 KD
PBS
PFF
wtTIDM
mTIDM
g
Iba1/Actin
h
iNOS/Actin
i
IL-1β/Actin
j
GFAP/Actin
ns

Figure S5. Suppression of microglial activation in midbrain of PFF-injected A53T mice by wtTIDM peptide. A53T mice (3 months old) were microinjected with 5 µg of PFF in both the hemispheres of brain (a). Following 2 months of surgery, animals received nasal treatment with either wild type TIDM (wtTIDM) or mutated TIDM (mTIDM) at the dose of 0.1 mg/kg body weight. After 1 month of TIDM treatment, behavioural analyses were performed, mice were sacrificed and biochemical experiments were conducted (b). Microglial activation in substantia nigra (SN) was monitored by double immunofluorescence analysis for Iba1 (red) and iNOS (green). Scale bar for lower (20x) and higher (60x) magnification images were 20 and 10 µm respectively. Two midbrain sections from each mouse brain of all the experimental groups were used for immunostaining and the number of microglia (c, d, p=0.00014 for A53T+PBS vs A53T+PFF and p=0.00009 for A53T+PFF vs A53T+PFF+wtTIDM) and microglia specific iNOS expression are shown (c, e, p=0.0008 for nTg vs A53T+PBS, p=0.0499 for A53T+PBS vs A53T+PFF and p=0.0013 for A53T+PFF vs A53T+PFF+wtTIDM). Protein levels of Iba1 (f, g, p=0.0087 for nTg vs A53T+PBS, p=0.0224 for A53T+PBS vs A53T+PFF and p=0.0167 for A53T+PFF vs A53T+PFF+wtTIDM), iNOS (f, h, p=0.0085 for nTg vs A53T+PBS, p=0.0024 for A53T+PBS vs A53T+PFF and p=0.0039 for A53T+PFF vs A53T+PFF+wtTIDM), IL-1β (f, i, p=0.0252 for A53T+PBS vs A53T+PFF and p=0.0074 for A53T+PFF vs A53T+PFF+wtTIDM), and GFAP (f, j, p=0.0086 for nTg vs A53T+PBS, p=0.039 for A53T+PBS vs A53T+PFF and p=0.0057 for A53T+PFF vs A53T+PFF+wtTIDM) in midbrain was assessed by immunoblotting. Relative protein expression is shown with respect to the loading control actin. One-way ANOVA followed by Tukey's multiple comparison tests was conducted for statistical analyses. $^*p< 0.05$, $^{**}p< 0.01$, $^{***}p< 0.001$ indicate significance compared to respective groups. Values are given as mean ± SEM (n=5 animals per group).

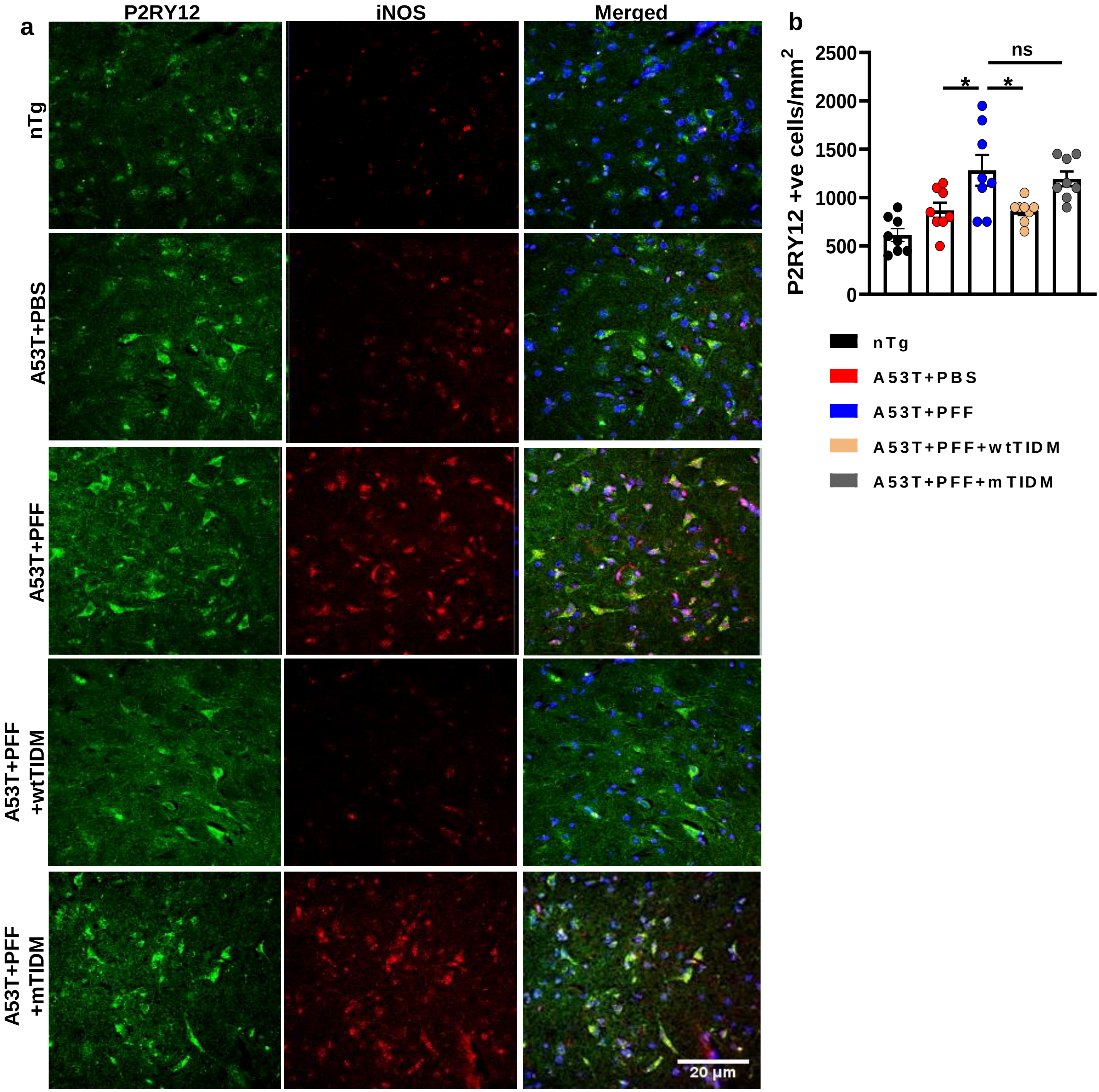

Figure S6. Inhibition of microgliosis in midbrain of PFF-injected A53T mice by wtTIDM peptide. Microgliosis in midbrain of PFF-induced A53T mice was measured by performing immunofluorescence analysis using microglia specific marker, P2RY12 (shown in green), co-stained with iNOS (red). Scale bar for lower (20x) and higher (60x) magnification images were 20 and 10 µm respectively (a, b, *p*=0.0258 for A53T+PBS vs A53T+PFF and *p*=0.0229 for A53T+PFF vs A53T+PFF+wtTIDM). Two midbrain sections from each mouse brain of all the experimental groups were used for immunostaining. One-way ANOVA followed by Tukey's multiple comparison tests was conducted for statistical analyses. $^{*}p < 0.05$ indicates significance compared to respective groups. Values are given as mean ± SEM (n=4 animals per group).

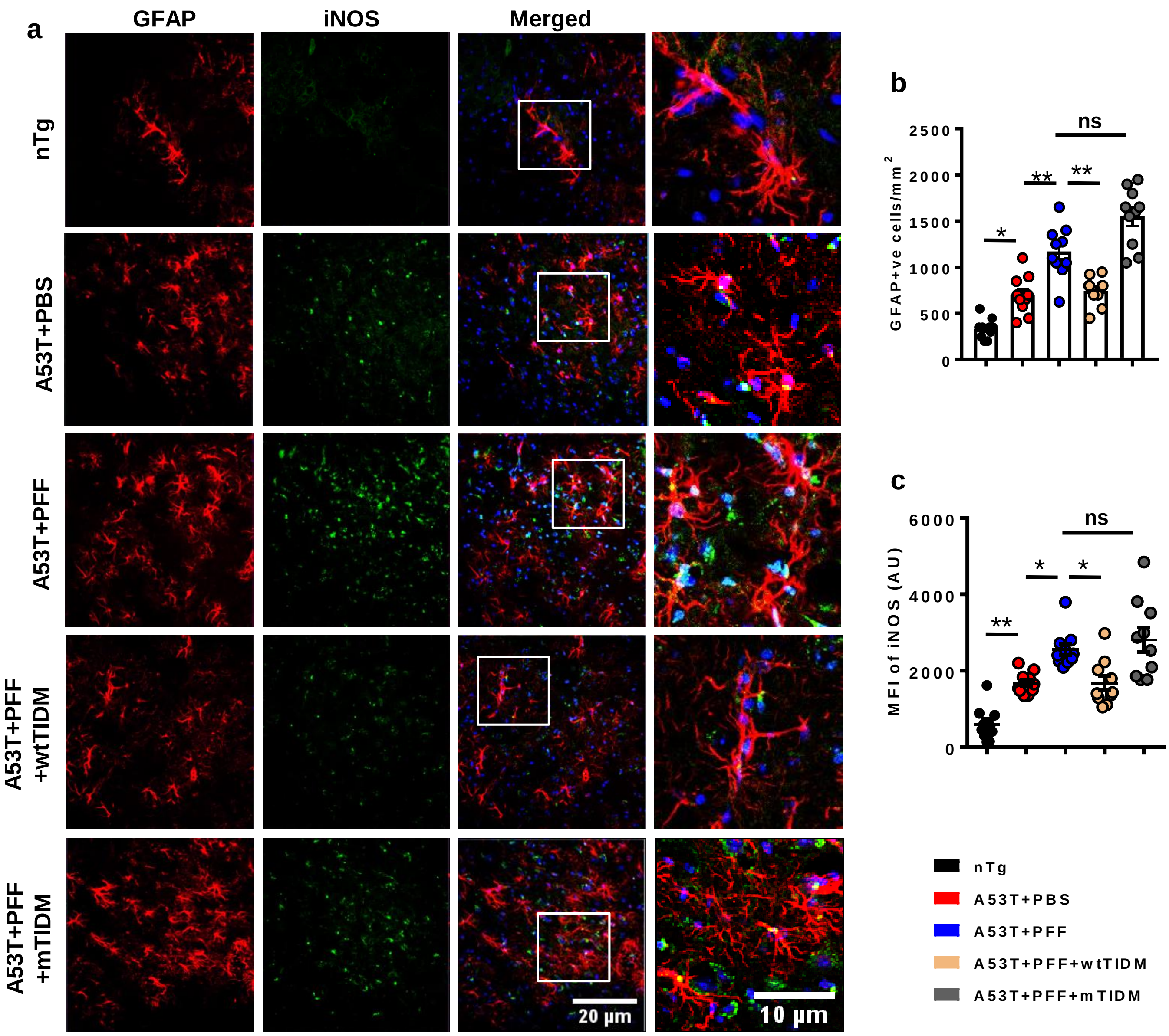

Figure S7. Prevention of astrogliosis in midbrain of PFF-injected A53T mice by wtTIDM. Astroglial activation in midbrain of preformed fibril (PFF)-seeded A53T mice is shown by double immunostaining for GFAP (red) and iNOS (green) (a). Number of astroglia (b, p=0.043 for nTg vs A53T+PBS, p=0.0034 for A53T+PBS vs A53T+PFF and p=0.0043 for A53T+PFF vs A53T+PFF+wtTIDM) in midbrain and MFI of astroglia specific iNOS expression (c, p=0.00182 for nTg vs A53T+PBS, p=0.0279 for A53T+PBS vs A53T+PFF and p=0.0151 for A53T+PFF vs A53T+PFF+wtTIDM) and were measured by ImageJ. Two midbrain sections from each mouse brain were used for the analyses. One-way ANOVA followed by Tukey's multiple comparison tests was conducted for statistical analyses. *p< 0.05, **p< 0.01, ***p< 0.001 indicate significance compared to respective groups. Values are given as mean ± SEM (n=5 animals per each group).

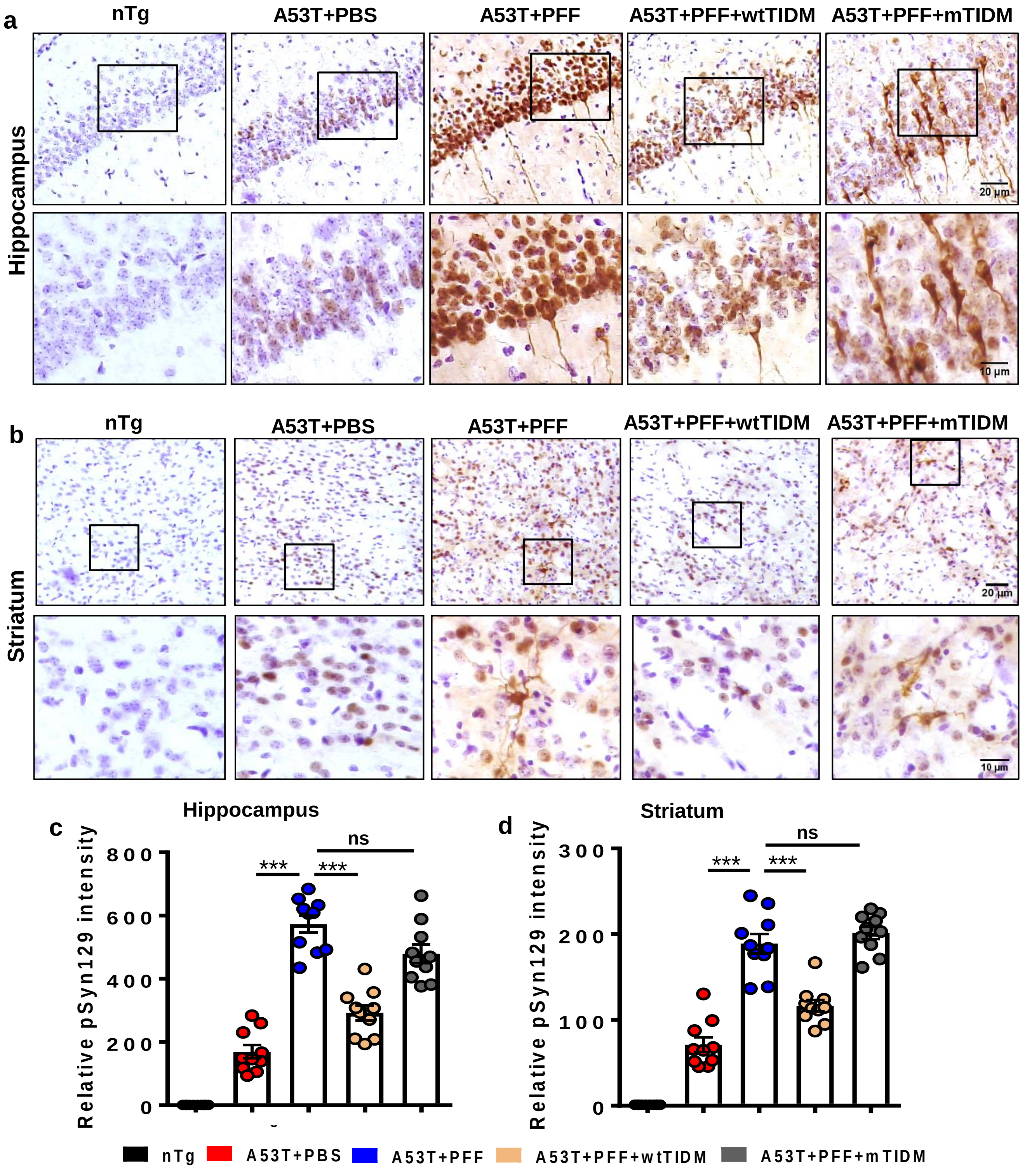

Figure S8. Prevention of α-syn spreading in hippocampus and striatum of PFF-seeded mice by wtTIDM peptide. Spreading of α-syn in hippocampus and striatum of PFF-seeded control and wtTIDM- or mTIDM-treated mice was monitored by immunohistochemistry for pathological phosphoserine 129 form of α-syn (pSyn129) (a, b). Two sections from each brain were used for the staining. Intensity of pSyn129 was assessed using the color deconvolution option followed by intensity measurement in the Fiji software (c, d, n=5 animals, p=0.00001 for A53T+PBS vs A53T+PFF and A53T+PFF vs A53T+PFF+wtTIDM). One-way ANOVA followed by Tukey's multiple comparison tests was conducted for statistical analyses. ***p< 0.001 indicate significance compared to respective groups. Values are given as mean ± SEM.

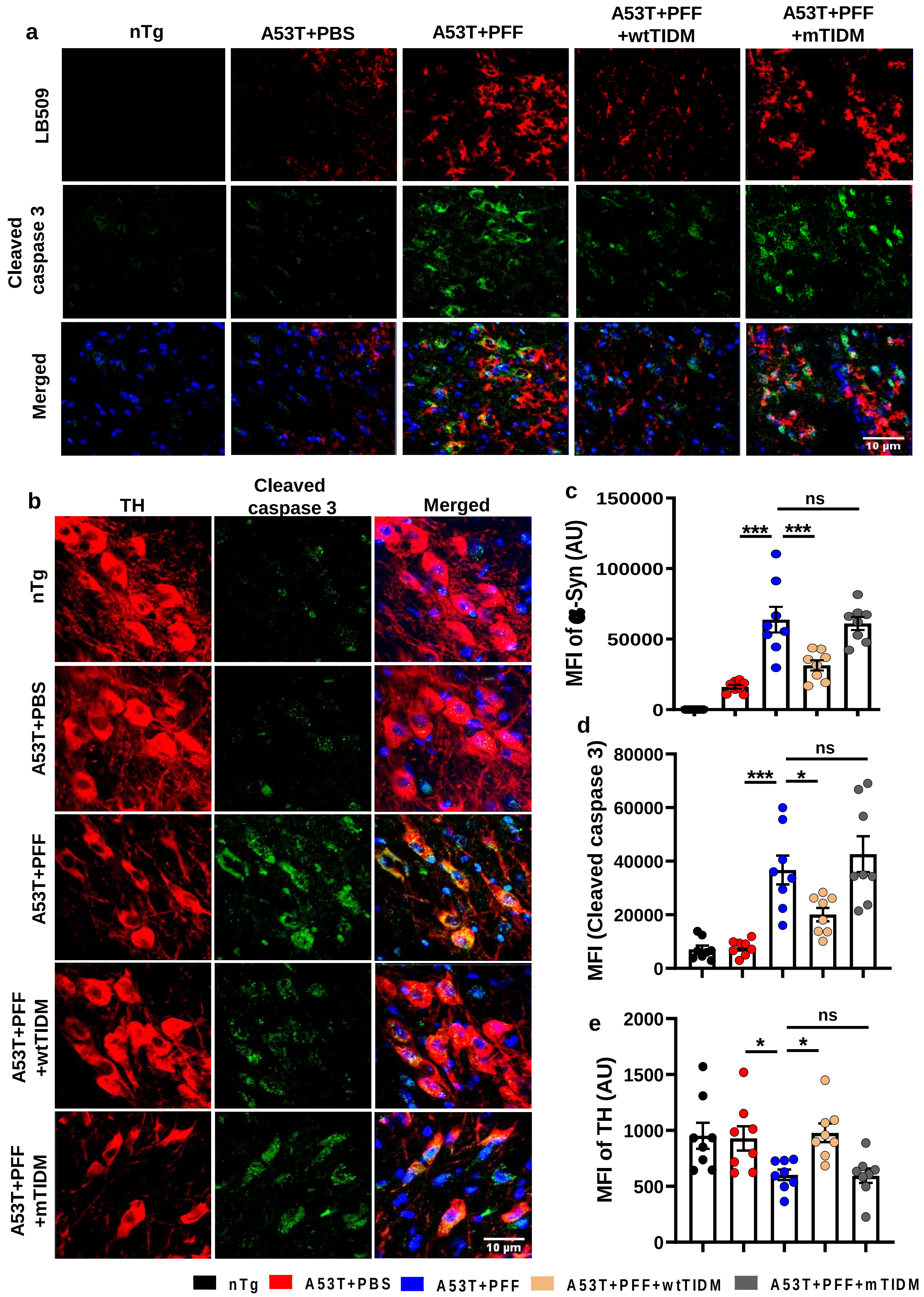
a
nTg
A53T+PBS
A53T+PFF
A53T+PFF +wtTIDM
A53T+PFF +mTIDM
LB509
Cleaved caspase 3
Merged
10 µm
b
TH
Cleaved caspase 3
Merged
nTg
A53T+PBS
A53T+PFF
A53T+PFF +wtTIDM
A53T+PFF +mTIDM
10 µm
c
MFI of α-Syn (AU)
150000
100000
50000
0
***
***
ns
d
MFI (Cleaved caspase 3)
80000
60000
40000
20000
0
***
*
ns
e
MFI of TH (AU)
2000
1500
1000
500
0
*
*
ns
nTg
A53T+PBS
A53T+PFF
A53T+PFF+wtTIDM
A53T+PFF+mTIDM

Figure S9. Inhibition of caspase 3 activation by wtTIDM in SN of PFF-seeded mice. Caspase 3 activation in nigral TH neurons was measured by conducting co-immunostaining of cleaved caspase 3 and aggregated α-syn in midbrain DAergic neurons in sections of all experimental groups (a). Cleaved caspase 3 expression in TH-positive cells was also evaluated by immunofluorescence analysis (b). Aggregated α-syn level (c, p=0.000001 for A53T+PBS vs A53T+PFF and p=0.00037 for A53T+PFF vs A53T+PFF+wtTIDM, cleaved caspase 3 expression (d, p=0.00013 for A53T+PBS vs A53T+PFF and p=0.047 for A53T+PFF vs A53T+PFF+wtTIDM) and TH expression (e, p=0.049 for A53T+PBS vs A53T+PFF and p=0.036 for A53T+PFF vs A53T+PFF+wtTIDM) in nigral sections were measured using ImageJ and the MFI values obtained from individual sections of animals are shown (c-e). For immunostaining 2 sections from each brain was considered. One-way ANOVA followed by Tukey's multiple comparison tests was conducted for statistical analyses. *p< 0.05, **p< 0.01, ***p< 0.001 indicate significance compared to respective groups. Values are given as mean ± SEM (n=4 animals per each group).

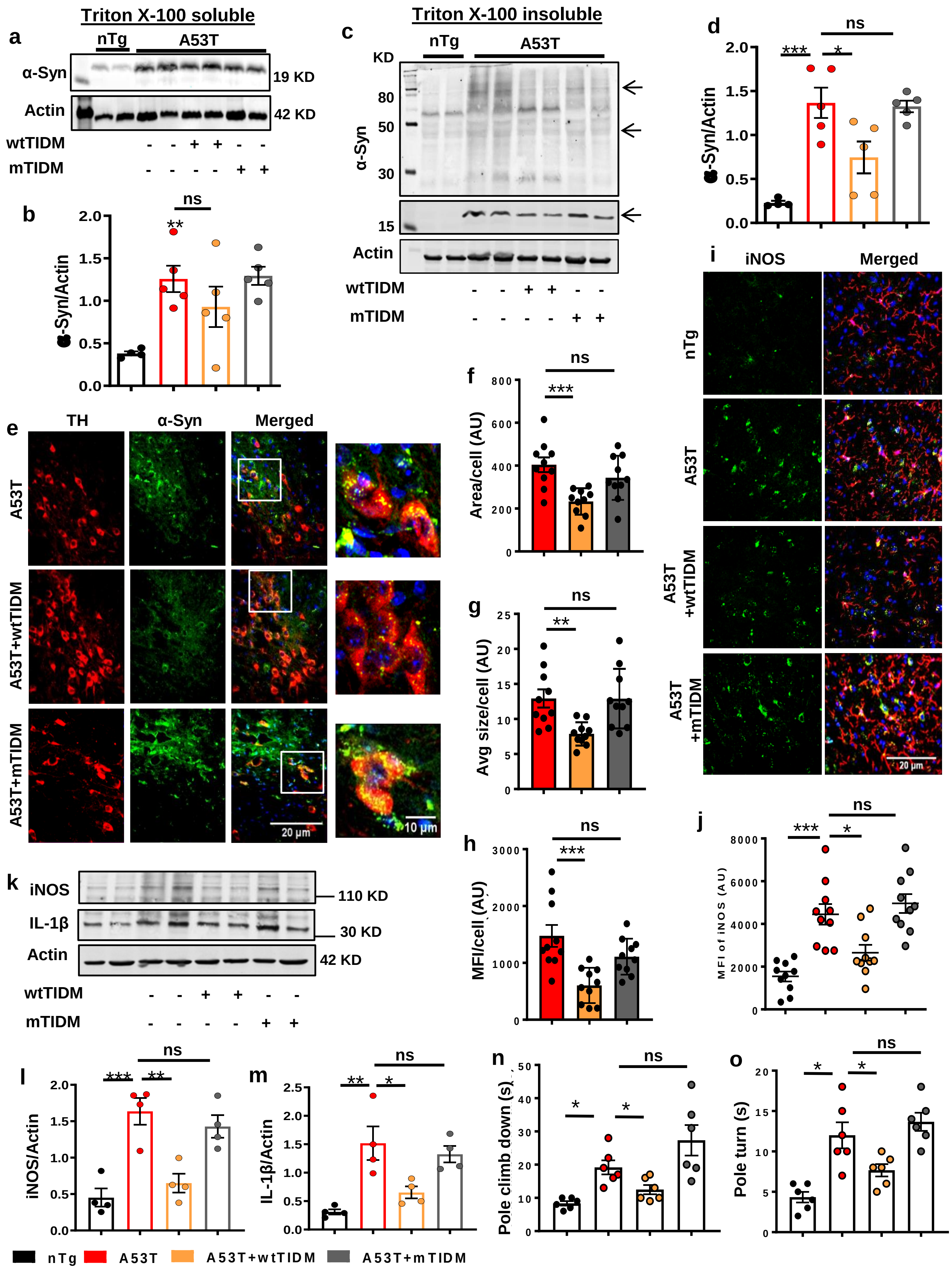

a
Triton X-100 soluble
nTg
A53T
α-Syn
19 KD
Actin
42 KD
wtTIDM
mTIDM
b
α-Syn/Actin
ns
**
c
Triton X-100 insoluble
KD
80
50
30
15
Actin
d
***
*
ns
e
TH
α-Syn
Merged
A53T
A53T+wtTIDM
A53T+mTIDM
20 μm
10 μm
f
Area/cell (AU)
g
Avg size/cell (AU)
h
MFI/cell (AU)
i
iNOS
Merged
nTg
A53T
A53T +wtTIDM
A53T +mTIDM
20 μm
j
MFI of iNOS (AU)
k
iNOS
110 KD
IL-1β
30 KD
Actin
42 KD
l
iNOS/Actin
m
IL-1β/Actin
n
Pole climb down (s)
o
Pole turn (s)
nTg
A53T
A53T+wtTIDM
A53T+mTIDM

Figure S10. Down-regulation of α-syn pathology by wtTIDM peptide in midbrain of 9 months old A53T mice. Eight-month old A53T animals were treated with 0.1 mg/kg/d wtTIDM or mTIDM for 1 month and then nigral level of α-syn was measured by immunoblotting in Triton X-100 soluble (a, b, n=4 for nTg and n=5 for A53T, A53T+wtTIDM, A53T+mTIDM, *p*=0.0097, nTg vs A53T) and insoluble fractions (c, d, n=4 for nTg and n=5 for A53T, A53T+wtTIDM, A53T+mTIDM, *p*=0.00024 nTg vs A53T, *p*=0.0236 A53T vs A53T+wtTIDM). Relative level of α-syn monomers in detergent soluble and insoluble fractions is presented with respect to actin. TH neuron specific α-syn expression was monitored by immunofluorescence. Scale bar for lower (20x) and higher (60x) magnification images are 20 and 10 µm respectively. Area (f, n=5, p=0.00094, for A53T vs A53T+wtTIDM) average size (g, n=5, *p*=0.0093 for A53T vs A53T+wtTIDM) and MFI (h, n=5, p=0.00038, for A53T vs A53T+wtTIDM) of α-syn aggregates in TH neurons were measured by ImageJ (e-h). Microglial activation was monitored by double-labelling for Iba1 and iNOS (i). Microglia specific iNOS intensity in each mouse brain of all the experimental groups is shown (j, n=5, *p*=0.000035 for nTg vs A53T and *p*=0.012 for A53T vs A53T+wtTIDM). Protein level of IL-1β (k, m, n=4, *p*=0.00185 for nTg vs A53T and *p*=0.0197 for A53T vs A53T+wtTIDM and iNOS (k, l, n=4, *p*=0.00059 for nTg vs A53T and *p*=0.0027 for A53T vs A53T+wtTIDM) in midbrain was measured by immunoblotting (k-m). Pole climb down time (n, n=6, *p*=0.04 for nTg vs A53T and *p*=0.044 for A53T vs A53T+wtTIDM) and pole turn time (o, n=6, *p*=0.031 for nTg vs A53T and *p*=0.042 for A53T vs A53T+wtTIDM) were monitored. One-way ANOVA followed by Tukey’s multiple comparison tests was conducted for statistical analyses. Values are given as mean ± SEM. **p*< 0.05, ***p*< 0.01, ****p*< 0.001.

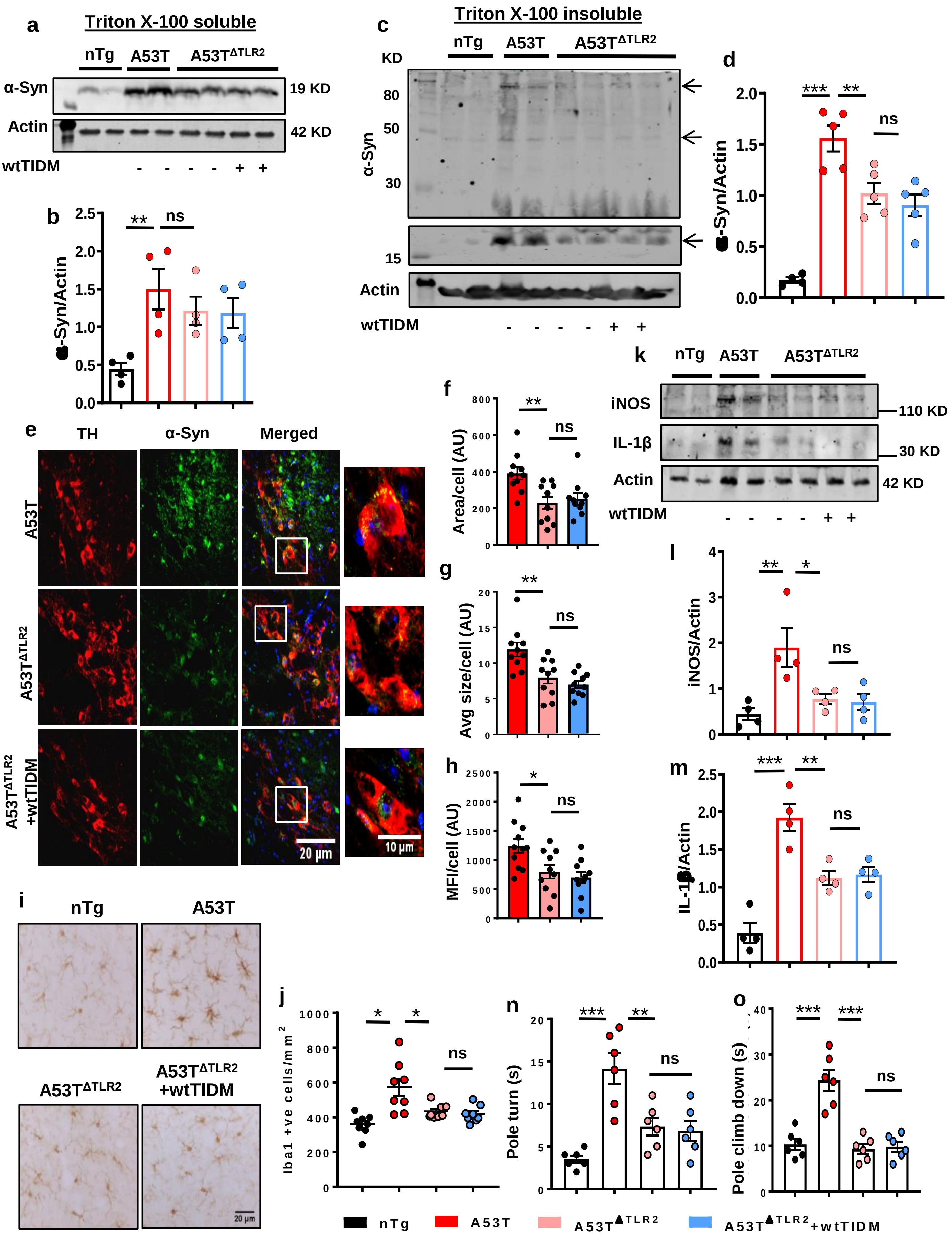

a
Triton X-100 soluble
nTg
A53T
A53T^ΔTLR2
α-Syn
19 KD
Actin
42 KD
wtTIDM
- - - - + +
b
α-Syn/Actin
** ns
c
Triton X-100 insoluble
nTg
A53T
A53T^ΔTLR2
KD
80
50
30
15
α-Syn
Actin
wtTIDM
- - - - + +
d
α-Syn/Actin
*** ** ns
e
TH
α-Syn
Merged
A53T
A53T^ΔTLR2
A53T^ΔTLR2 +wtTIDM
20 μm
10 μm
f
Area/cell (AU)
** ns
g
Avg size/cell (AU)
** ns
h
MFI/cell (AU)
* ns
i
nTg
A53T
A53T^ΔTLR2
A53T^ΔTLR2 +wtTIDM
20 μm
j
Iba1 +ve cells/mm2
* * ns
k
nTg
A53T
A53T^ΔTLR2
iNOS
110 KD
IL-1β
30 KD
Actin
42 KD
wtTIDM
- - - - + +
l
iNOS/Actin
** * ns
m
IL-1β/Actin
*** ** ns
n
Pole turn (s)
*** ** ns
o
Pole climb down (s)
*** *** ns
nTg
A53T
A53T^ΔTLR2
A53T^ΔTLR2+wtTIDM

Figure S11. Genetic knockdown of TLR2 reduces α-syn pathology in aged A53T mice. Western blot shows α-syn level in Triton X-100 soluble (a, b, n=4, *p*=0.0094 for nTg vs A53T) and insoluble (c, d, n=4 for nTg and n=5 for A53T, A53T$^{\Delta TLR2}$ and A53T$^{\Delta TLR2}$+wtTIDM, *p*=0.0001 for nTg vs A53T and *p*=0.0095 for A53T vs A53T$^{\Delta TLR2}$) fractions isolated from SN of nTg, A53T, A53T$^{\Delta TLR2}$ and A53T$^{\Delta TLR2}$+wtTIDM mice (a-d). Double immunostaining of α-syn (green) and TH (red) demonstrates presence of α-syn in DAergic neurons of SN of experimental mice (e). Area (f, n=5, *p*=0.0049 for A53T vs A53T$^{\Delta TLR2}$), average size (g, n=5, *p*=0.0045 for A53T vs A53T$^{\Delta TLR2}$) and MFI (h, n=5, *p*=0.0258 for A53T vs A53T$^{\Delta TLR2}$) of α-syn aggregates in TH neurons of SN were measured by ImageJ (f-h). Microglial number in midbrain of nTg, 9-month old A53T and A53T$^{\Delta TLR2}$ mice was evaluated by Iba1 immunostaining in midbrain sections. For the experiment 2 sections per brain were used and number of Iba1-positive microglia was counted by ImageJ (i, j, n=4, *p*=0.0114 for nTg vs A53T and *p*=0.0142 for A53T vs A53T$^{\Delta TLR2}$). Protein level of IL-1β (k, m, n=4, *p*=0.0001 for nTg vs A53T and *p*=0.0042 for A53T vs A53T$^{\Delta TLR2}$) and iNOS (k, l, n=4, *p*=0.0054 for nTg vs A53T and *p*=0.0299 for A53T vs A53T$^{\Delta TLR2}$) in midbrain was measured by immunoblotting and relative protein expression is shown with respect to actin (k-m). Pole turn time (n, n=6, *p*=0.0001 for nTg vs A53T and *p*=0.0037 for A53T vs A53T$^{\Delta TLR2}$) and climb down time (o, n=6, *p*=0.0001 for nTg vs A53T and *p*=0.0001 for A53T vs A53T$^{\Delta TLR2}$) are shown. One-way ANOVA followed by Tukey's multiple comparison tests was conducted for statistical analyses. *p< 0.05, **p< 0.01, ***p< 0.001 indicate significance compared to respective groups. Values are given as mean ± SEM.

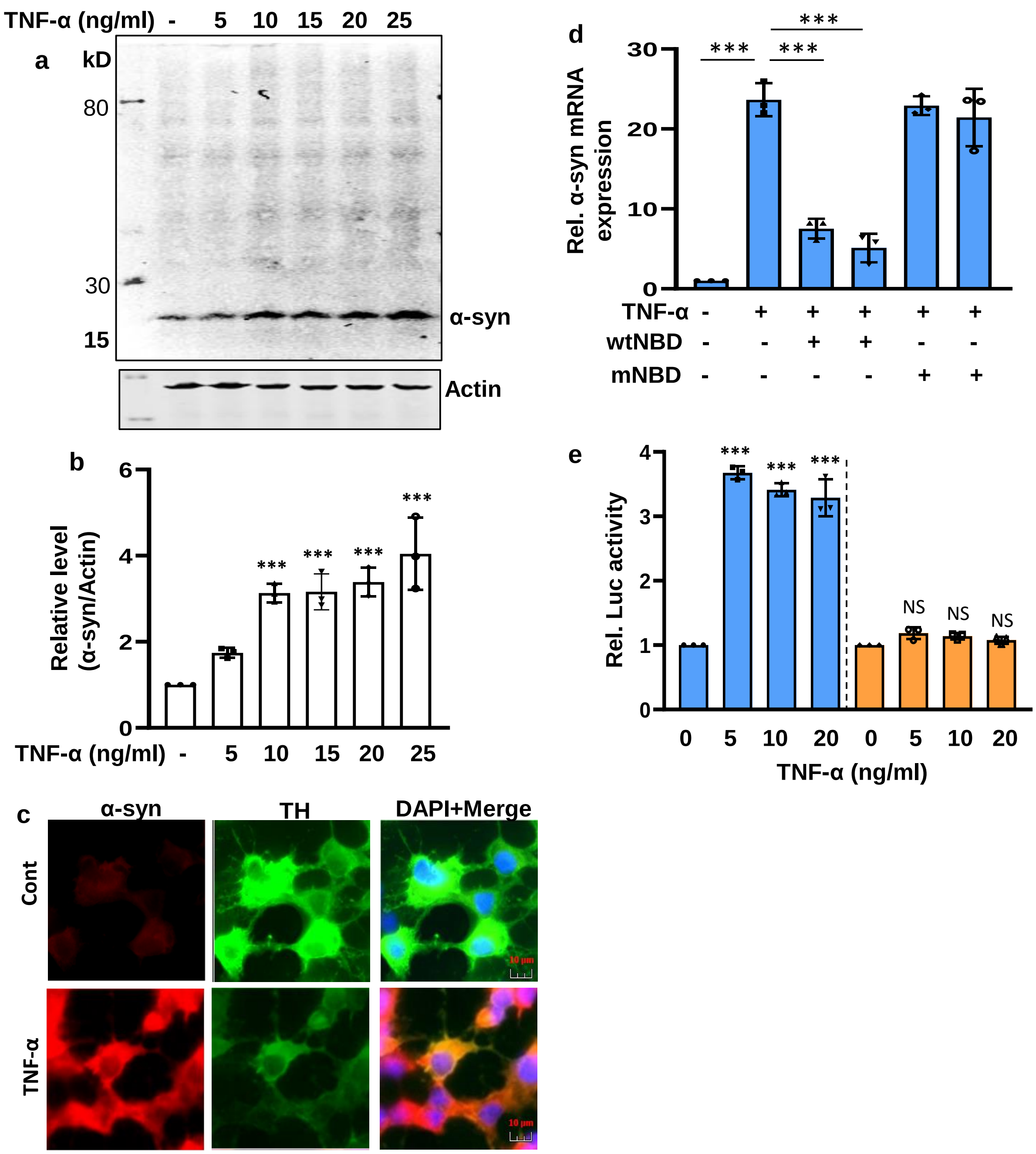

Figure S12. TNFα upregulates α-syn in MN9D cells via NF-κB. Cells were stimulated with different concentrations of TNF-α under serum-free conditions. After 12 h of TNF-α treatment, the protein level of α-syn was examined by Western blot (a and b, p=0.0005 control vs TNFα 10 ng/ml, p=0.0004 control vs TNFα 15 ng/ml, p=0.0002 control vs TNFα 20 ng/ml, p=0.00001 control vs TNFα 25 ng/ml). After 12 h of TNF-α treatment, cells were also immunostained with antibodies against α-syn and TH (c). Cells, pre-incubated with either wtNBD peptide or mNBD peptide for 30 min, were stimulated by TNF-α for 4 h followed by analysis of α-syn mRNAs by quantitative real-time PCR (d, p=0.0001 control vs TNFα, p=0.0001 TNFα vs wtNBD). MN9D cells were transfected with pα-syn(WT)-Luc and pα-syn(Mut)-Luc for 24 h followed by treatment with TNF-α and subjected to luciferase assay (e, p=0.00001 for control vs TNFα all doses). One-way ANOVA followed by Tukey's multiple comparison tests was conducted for statistical analyses. ***, p< 0.001versus untreated control. NS, not significant with respect to untreated pα-syn(Mut)-Luc transfected control. All results are mean ± S.D. of at least three independent experiments (n = 3).

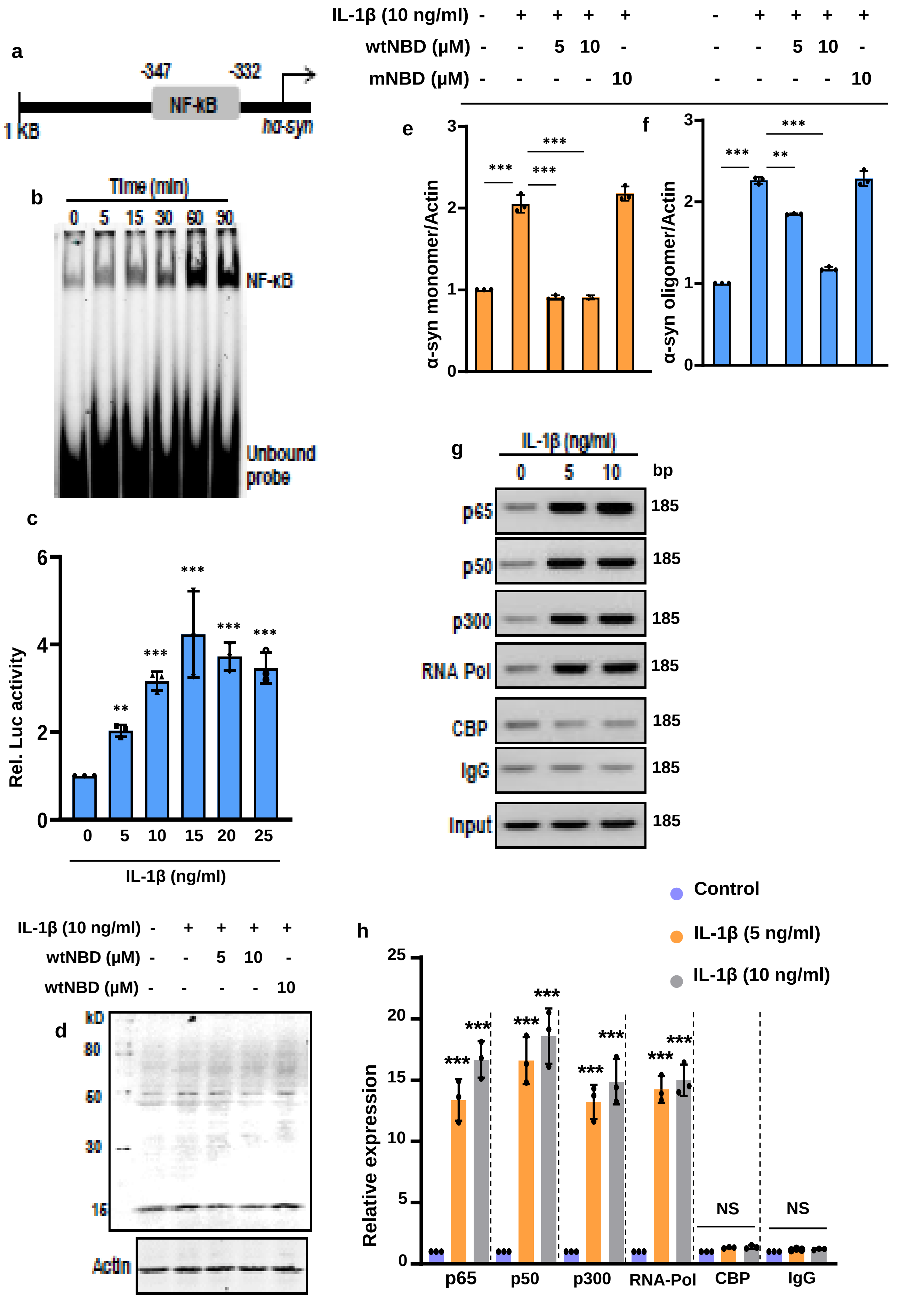
a
-347
-332
NF-kB
1 KB
hα-syn
b
Time (min)
0 5 15 30 60 90
NF-κB
Unbound probe
c
Rel. Luc activity
IL-1β (ng/ml)
0 5 10 15 20 25
IL-1β (10 ng/ml) - + + + +
wtNBD (μM) - - 5 10 -
wtNBD (μM) - - - - 10
d
kD
80
60
30
16
Actin
IL-1β (10 ng/ml) - + + + + - + + + +
wtNBD (μM) - - 5 10 - - - 5 10 -
mNBD (μM) - - - - 10 - - - - 10
e
α-syn monomer/Actin
f
α-syn oligomer/Actin
g
IL-1β (ng/ml)
0 5 10
bp
p65
p50
p300
RNA Pol
CBP
IgG
Input
185
h
Control
IL-1β (5 ng/ml)
IL-1β (10 ng/ml)
Relative expression
NS
p65
p50
p300
RNA-Pol
CBP
IgG

Figure S13. IL-1β induces α-syn expression in human SH-SY5Y neuronal cells via NF-κB. (a) The presence of NF-κB binding site in human α-syn gene. Human SH-SY5Y cells were incubated with IL-1β for different time periods followed by monitoring the activation of NF-κB by EMSA (b). Cells were transfected with PBIIx-Luc for 24 h followed by treatment with different concentrations of IL-1β and subjected to luciferase assay (c, p=0.034 control vs IL-1β 5 ng/ml, p=0.001 control vs IL-1β 10 ng/ml, p=0.0006 control vs IL-1β 15 ng/ml, p=0.0001 control vs IL-1β 20 ng/ml, p=0.0003 control vs IL-1β 25 ng/ml). Cells were treated with wtNBD or mNBD peptides and for 30 min followed by stimulation with different concentrations of IL-1β under serum-free conditions for 12 h and then α-syn protein level was measured by Western blot (d, e, f, p=0.00001 for α-syn monomer, control vs IL-1β, p=0.000006 for IL-1β vs wtNBD, for α-syn oligomer, p=0.000062 control vs IL-1β). Immunoprecipitated chromatin fragments were amplified by semi-quantitative (g) and real-time PCR (h, p65, p=0.000061 for control vs IL-1β 5 ng/ml and p=0.000015 for control vs IL-1β 10 ng/ml; p50, p=0.000076 for control vs IL-1β 5 ng/ml and p=0.000038 for control vs IL-1β 10 ng/ml; p300, p=0.000078 for control vs IL-1β 5 ng/ml and p=0.000038 for control vs IL-1β 10 ng/ml; RNA-Pol, p=0.000066 for control vs IL-1β 5 ng/ml and p=0.000047 for control vs IL-1β 10 ng/ml) for the region spanning the NF-κB binding site of the human α-syn promoter using primers mentioned under “Materials and Methods”. One-way ANOVA followed by Tukey’s multiple comparison tests was conducted for statistical analyses. ** and *** indicate $p < 0.01$ and $p < 0.001$ versus untreated control. Results are the mean ± S.D. of three separate experiments (n = 3).

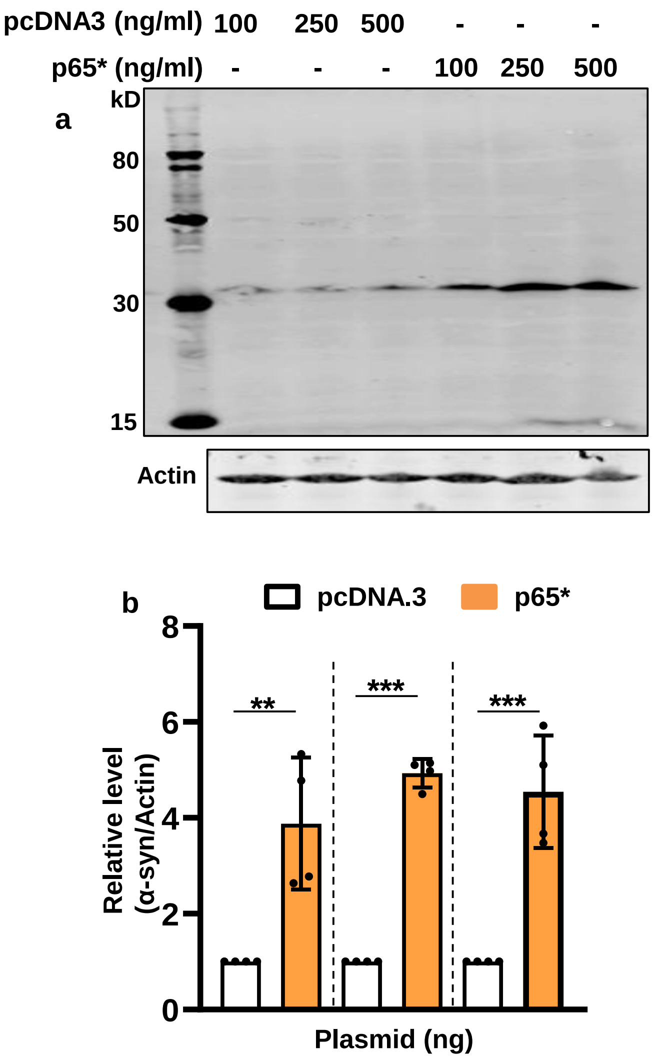

Figure S14. Effect of over expression of wild-type p65 (*p65) on α-syn protein production in mouse MN9D cells. MN9D plated in 12-well plates were transfected with either different concentrations of either of pcDNA3 or wild type p65 for 24 h and cell lysed were analyzed by western blot (a, b, *p*=0.0057 pcDNA.3 vs p65 100 ng/ml, *p*=0.00001 pcDNA.3 vs p65 250 ng/ml, *p*=0.00092 pcDNA.3 vs p65 500 ng/ml). Two-tailed unpaired t-test was performed to determine statistical significance between p65 and corresponding empty vector transfected groups. ** and *** indicate $p < 0.01$ and $p < 0.001$ versus untreated control. Results are the mean ± S.D. of three separate experiments (n = 3).

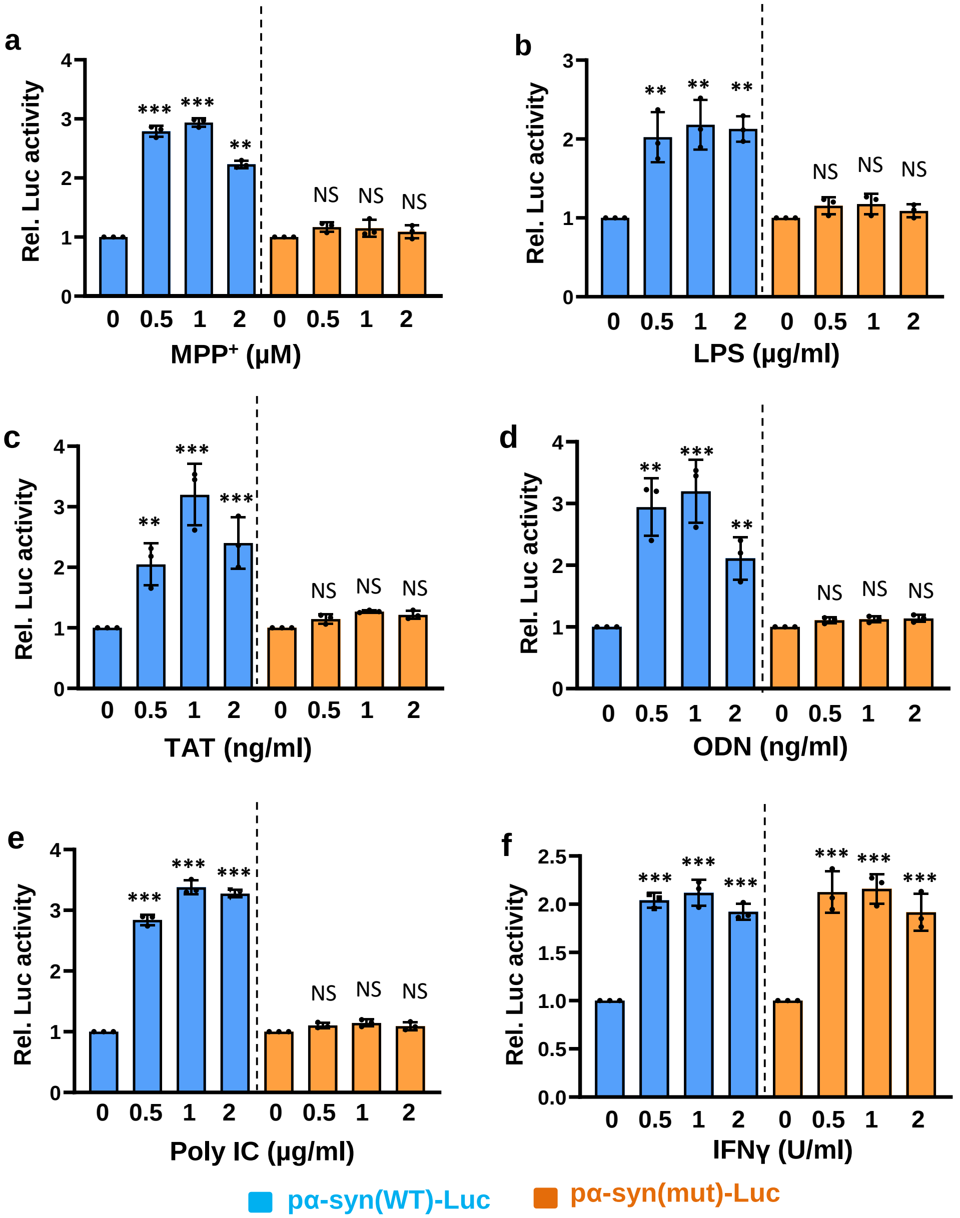

Figure S15. NF-κB-mediated α-syn promoter activation by different stimuli. MN9D cells were transfected with pα-syn(WT)-Luc and pα-syn(Mut)-Luc for 24 h followed by treatment with different stimuli including MPP^+ (a, p=0.000006 control vs MPP^+ 0.5 μM, p=0.000004 control vs MPP^+ 1 μM, p=0.0000049 control vs MPP^+ 2 μM), LPS (b, p=0.00334 control vs LPS 0.5 μg/ml, p=0.00133 control vs LPS 1 μg/ml, p=0.00179 control vs LPS 2 μg/ml), Tat (c, p=0.0036 control vs Tat 0.5 ng/ml, p=0.00042 control vs Tat 1 ng/ml, p=0.0077 control vs Tat 2 ng/ml), ODN (d, p=0.0012 control vs ODN 0.5 ng/ml, p=0.00052 control vs ODN 1 ng/ml, p=0.0031 control vs ODN 2 ng/ml), poly IC (e, p=0.000001 control vs poly IC 0.5 μg/ml, p=0.000001 control vs poly IC 1 μg/ml, p=0.000001 control vs poly IC 2 μg/ml) and IFN-γ (f, p=0.0000025 control vs IFN-γ 0.5 U/ml, p=0.0000014 control vs IFN-γ 1 U/ml, p=0.0000062 control vs IFN-γ 2 U/ml). One-way ANOVA followed by Tukey's multiple comparison tests was conducted separately among groups referring to pα-syn(WT)-Luc and pα-syn(Mut)-Luc transfected cells. ** and *** indicate $p < 0.01$ and $p < 0.001$ versus untreated control. Results are represented as mean ± S.D. of three separate experiments (n = 3).

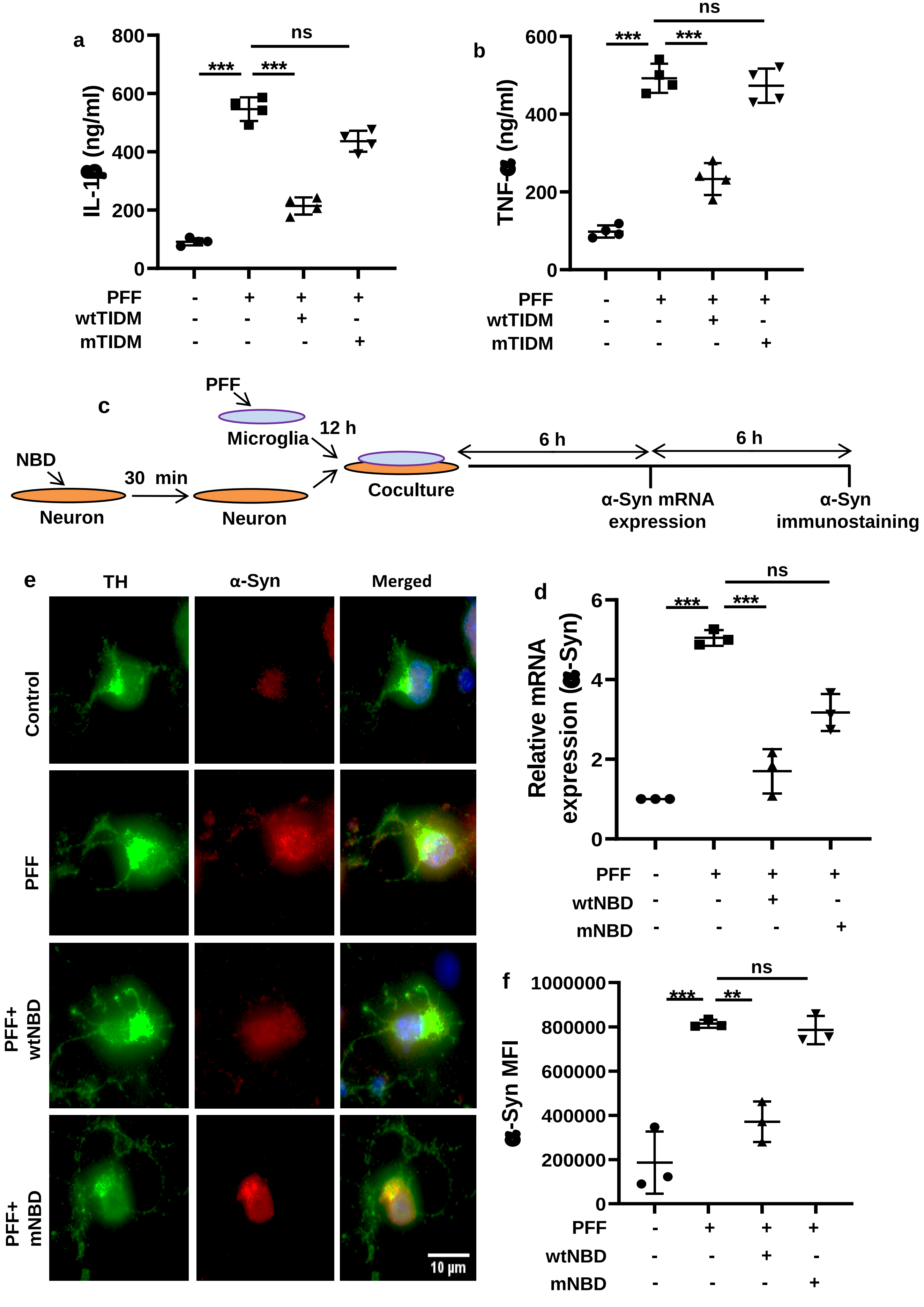

a
IL-1 (ng/ml)
800
600
400
200
0
***
***
ns
PFF - + + +
wtTIDM - - + -
mTIDM - - - +
b
TNF- (ng/ml)
600
400
200
0
***
***
ns
PFF - + + +
wtTIDM - - + -
mTIDM - - - +
c
PFF
NBD
Microglia
12 h
30 min
Neuron
Neuron
Coculture
6 h
6 h
α-Syn mRNA expression
α-Syn immunostaining
e
TH
α-Syn
Merged
Control
PFF
PFF+ wtNBD
PFF+ mNBD
10 µm
d
Relative mRNA expression
6
4
2
0
***
***
ns
PFF - + + +
wtNBD - - + -
mNBD - - - +
f
-Syn MFI
1000000
800000
600000
400000
200000
0
***
**
ns
PFF - + + +
wtNBD - - + -
mNBD - - - +

Figure S16. Inhibition of NF-κB activation attenuates inflammatory molecule-induced α-syn up-regulation in primary DAergic neurons. Induction of inflammatory molecules by PFF in primary microglia was assessed by measuring the level of IL-1β (a, n=3 separate samples, p=0.00014 control vs PFF, p=0.0002 PFF vs wtTIDM+PFF) and TNF-α (b, n=3 separate samples, p=0.00002 control vs PFF, p=0.000017 PFF vs wtTIDM+PFF) from the spent media following 24 h of PFF treatment. Primary microglia was treated with PFF for 12 h and following that the microglia was co-cultured with primary DAergic neurons, which were treated with wtNBD or mNBD peptides (5 µM) 30 min prior to co-culturing (c). The mRNA expression of α-syn was measured from the DAergic neurons after 6 h of co-culturing (d, n=3 separate samples, p=0.00048 control vs PFF, p=0.0002 PFF vs wtTIDM+PFF). Protein expression of α-syn was evaluated by co-immunostaining of TH and α-syn in primary neurons following 12 h of co-culturing and the expression of α-syn was measured using ImageJ (e, f, n=3 separate experiments, p=0.0001 control vs PFF, p=0.0014 PFF vs wtTIDM+PFF). One-way ANOVA followed by Tukey's multiple comparison tests was conducted for statistical analyses. ** and *** indicate $p < 0.01$ and $p < 0.001$ compared to respective groups. Results are represented as mean ± S.D. of three separate experiments.

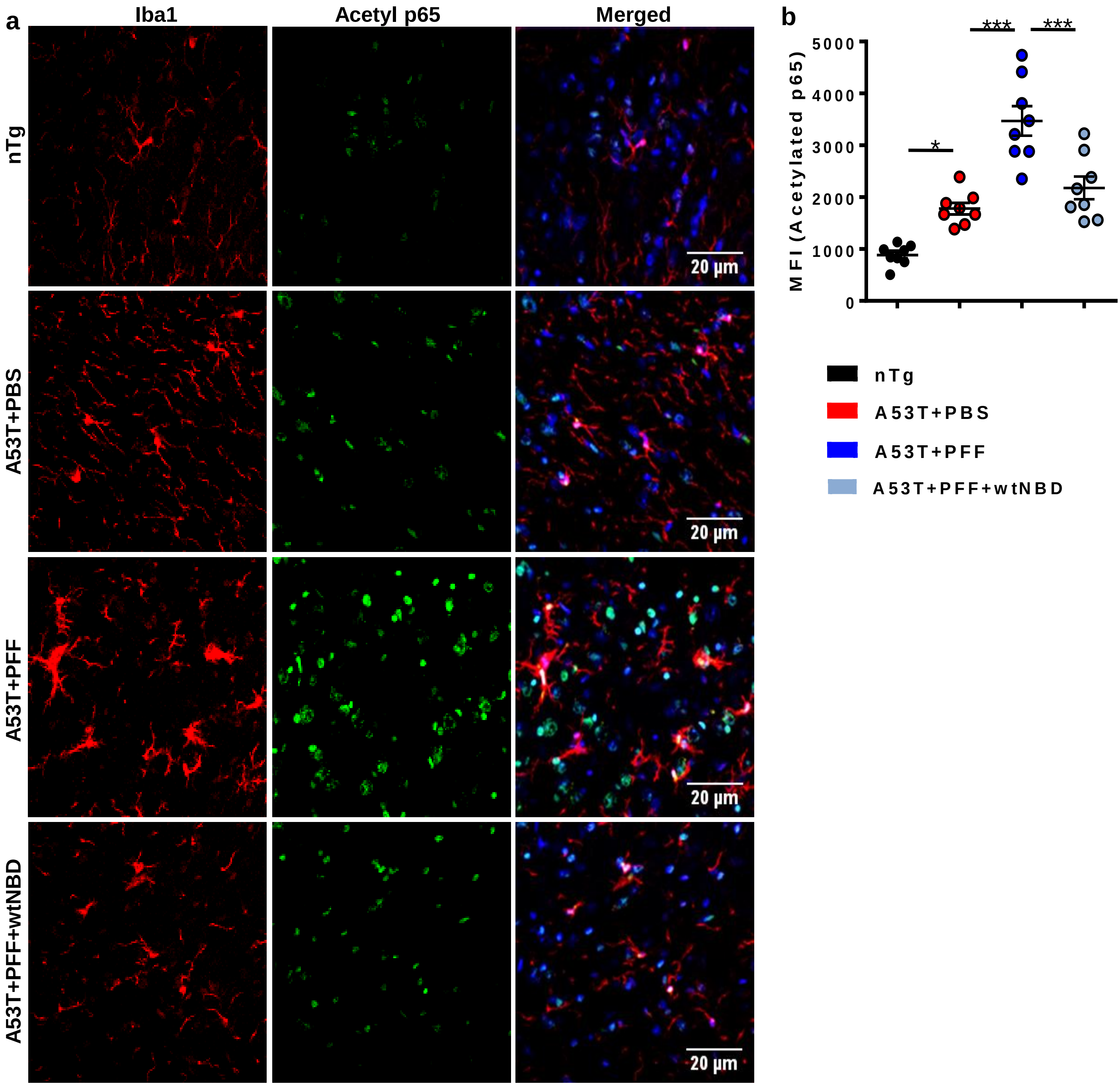

Figure S17. The wtNBD peptide inhibits NF-κB activation in SN of PFF-induced brain. A53T mice were seeded with PFF bilaterally and following 2 months of brain surgery, animals were given intranasal administration of 0.1 mg/kg wtNBD peptide. Activation of NF-κB in SN was monitored by evaluating the level of acetylated (K310) p65 in Iba1+ve microglia in different groups of mice (a, b, p=0.0138 nTg vs A53T+PBS, p=0.0005 A53T+PBS vs A53T+PFF and p=0.0003 A53T+PFF vs A53T+PFF+wtNBD). Statistical significance was determined by one-way ANOVA followed by Tukey's multiple comparison tests. *$p< 0.05$, ***$p< 0.001$ indicate significance compared to respective groups. Values are given as mean ± SEM (n=4 animals per group).

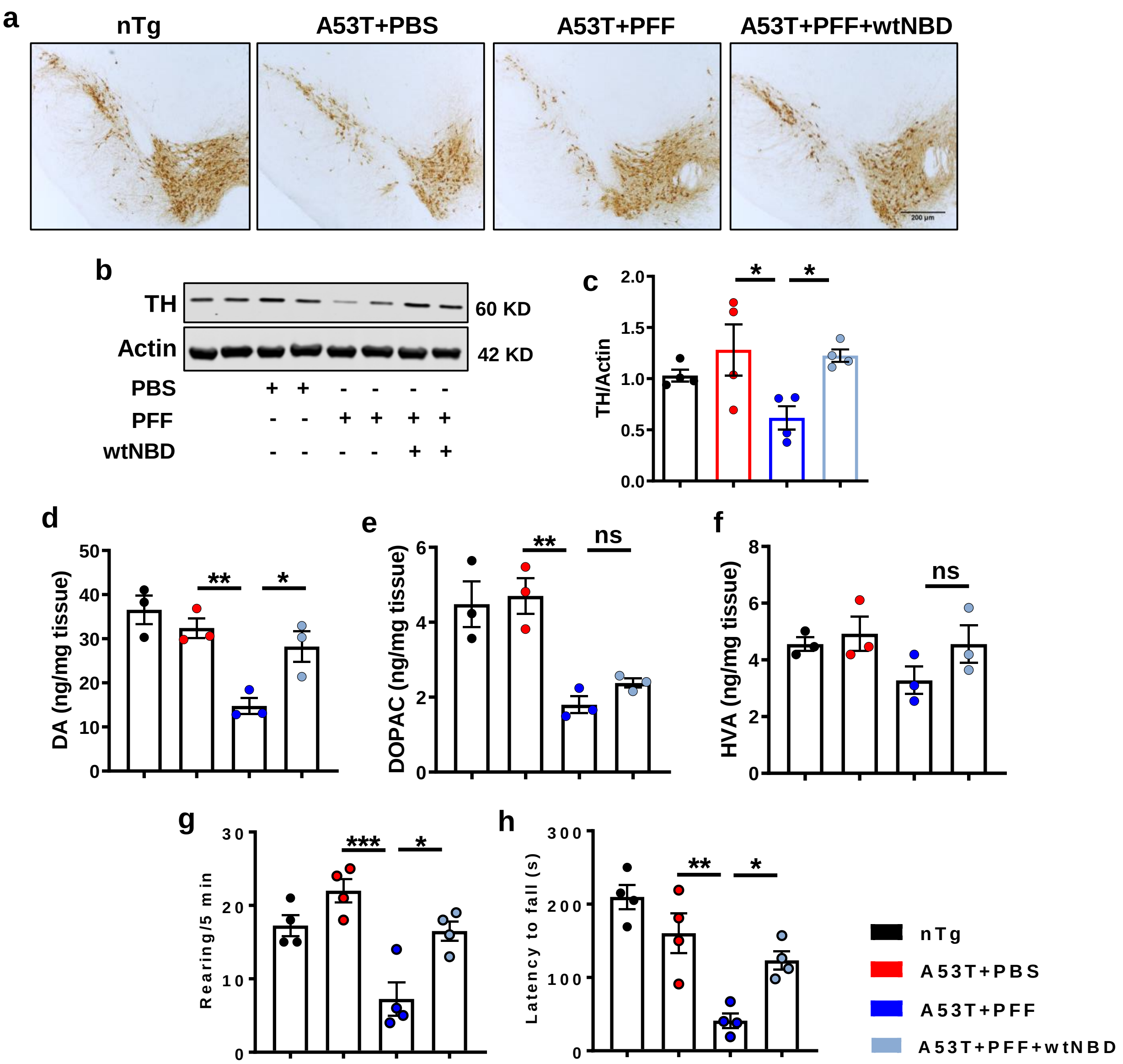

Figure S18. The wtNBD peptide attenuates PFF-induced parkinsonian pathology in A53T mice. PFF-seeded A53T animals were given intranasal administration of 0.1 mg/kg wtNBD peptide. Parkinsonian pathology was evaluated by TH immunohistochemistry of nigral sections (a), immunoblotting of total TH level in SN (b and c, n=4, p=0.03 A53T+PBS vs A53T+PFF and p=0.048 A53T+PFF vs A53T+PFF+wtNBD), assessing striatal level of dopamine (DA, n=3, p=0.0088 A53T+PBS vs A53T+PFF and p=0.037 A53T+PFF vs A53T+PFF+wtNBD), and its metabolites 3,4-dihydroxyphenyl acetate (DOPAC, n=3, p=0.0074 A53T+PBS vs A53T+PFF), homovanillic acid (HVA, n=3) (d-f). Motor behavioral analyses of animals were performed by rearing test (g, n=4, p=0.0003 A53T+PBS vs A53T+PFF and p=0.0107 A53T+PFF vs A53T+PFF+wtNBD) and rotarod test (h, n=4, p=0.0023 A53T+PBS vs A53T+PFF and p=0.0297 A53T+PFF vs A53T+PFF+wtNBD). Statistical significance was determined by one-way ANOVA followed by Tukey's multiple comparison tests. *p< 0.05, **p< 0.01, ***p< 0.001 indicate significance compared to respective groups. Values are given as mean ± SEM.
