# Supplementary material for: Selective targeting of the TLR2/MyD88/NF-κB pathway reduces α-synuclein spreading in vitro and in vivo
Source: Nat Commun. 2021 Sep 10;12:5382. doi: 10.1038/s41467-021-25767-1 (PMC8433339; doi:10.1038/s41467-021-25767-1)
Supplement: Supplementary file 3 — Reporting Summary [file 41467_2021_25767_MOESM3_ESM.pdf]

nature research

Corresponding author(s): Kalipada Pahan

Last updated by author(s): 2021/08/06

Reporting Summary

Nature Research wishes to improve the reproducibility of the work that we publish. This form provides structure for consistency and transparency in reporting. For further information on Nature Research policies, see our Editorial Policies and the Editorial Policy Checklist.

Statistics

For all statistical analyses, confirm that the following items are present in the figure legend, table legend, main text, or Methods section.

n/a	Confirmed	
☐	☒	The exact sample size (*n*) for each experimental group/condition, given as a discrete number and unit of measurement
☐	☒	A statement on whether measurements were taken from distinct samples or whether the same sample was measured repeatedly
☐	☒	The statistical test(s) used AND whether they are one- or two-sided<br>*Only common tests should be described solely by name; describe more complex techniques in the Methods section.*
☒	☐	A description of all covariates tested
☐	☒	A description of any assumptions or corrections, such as tests of normality and adjustment for multiple comparisons
☐	☒	A full description of the statistical parameters including central tendency (e.g. means) or other basic estimates (e.g. regression coefficient) AND variation (e.g. standard deviation) or associated estimates of uncertainty (e.g. confidence intervals)
☐	☒	For null hypothesis testing, the test statistic (e.g. *F*, *t*, *r*) with confidence intervals, effect sizes, degrees of freedom and *P* value noted<br>*Give P values as exact values whenever suitable.*
☒	☐	For Bayesian analysis, information on the choice of priors and Markov chain Monte Carlo settings
☒	☐	For hierarchical and complex designs, identification of the appropriate level for tests and full reporting of outcomes
☒	☐	Estimates of effect sizes (e.g. Cohen's *d*, Pearson's *r*), indicating how they were calculated

Our web collection on statistics for biologists contains articles on many of the points above.

Software and code

Policy information about availability of computer code

Data collection	Behavioral data on open field tests were collected by Noldus system and EthoVisionXT 11.5 software. Ct values of genes in real time PCR were collected by ABI-Prism7700 sequence detection system. Fluorescent images were collected by Olympus BX41 fluorescence microscope and Zeiss confocal microscope. Electron microscope images was obtained by Erlangshen ES1000W model 785 CCD camera and Digital Micrograph software (Version 1.7). Western blot bands were scanned by Lycor Odyssey infrared scanner. Other data were not collected by any softwares.
Data analysis	Image analysis was done by ImageJ (v.1.x) and Fiji (ImageJ2).<br>Band densities of Western blots were measured by ImageJ (v.1.x).<br>Counting of neurons was done by Stereo Investigator software.<br>Statistical analyses were conducted by GraphPad v.7.04

For manuscripts utilizing custom algorithms or software that are central to the research but not yet described in published literature, software must be made available to editors and reviewers. We strongly encourage code deposition in a community repository (e.g. GitHub). See the Nature Research guidelines for submitting code & software for further information.

Data

Policy information about availability of data

All manuscripts must include a data availability statement. This statement should provide the following information, where applicable:

- Accession codes, unique identifiers, or web links for publicly available datasets
- A list of figures that have associated raw data
- A description of any restrictions on data availability

Provide your data availability statement here.

April 2020

Field-specific reporting

Please select the one below that is the best fit for your research. If you are not sure, read the appropriate sections before making your selection.

- [x] Life sciences
- [ ] Behavioural & social sciences
- [ ] Ecological, evolutionary & environmental sciences

For a reference copy of the document with all sections, see nature.com/documents/nr-reporting-summary-flat.pdf

Life sciences study design

All studies must disclose on these points even when the disclosure is negative.

Sample size	In vitro experiments were performed independently at least three times as three independent repeats gave a P value less than 0.05. For mouse experiments, power calculation shows that 5 mice per group provides sufficient statistical power to achieve an alpha = 0.05 and beta<0.2 (power>80%). Therefore, in vivo experiments were performed with 5-6 mice per group.
Data exclusions	Data exclusion was not performed in the present study. All the obtained data from each experiments were considered.
Replication	In vitro studies included in the study were performed in at least three biological replicates. In vitro experiments were conducted by different researchers based on the need of reproducibility. All attempts at replication were successful. In vivo experiments were conducted with 5-6 different animals in each individual group.
Randomization	Animals were randomly allocated in each group irrespective of the sex or body weight. Only the proper age-matched animals (non-trasgenic and transgenic) were used for each experiment. For in vitro experiments, cells (BV-2, MN9D, primary microglia) were maintained in similar culture conditions. Control or synuclein-treated cells were plated in same number in culture wells for different experiments.
Blinding	Investigators were blinded to group allocation and data analysis. Investigators knew only about ID of each sample/mouse.

Reporting for specific materials, systems and methods

We require information from authors about some types of materials, experimental systems and methods used in many studies. Here, indicate whether each material, system or method listed is relevant to your study. If you are not sure if a list item applies to your research, read the appropriate section before selecting a response.

Materials & experimental systems

n/a	Involved in the study	
[ ]	[x]	Antibodies
[ ]	[x]	Eukaryotic cell lines
[x]	[ ]	Palaeontology and archaeology
[ ]	[x]	Animals and other organisms
[x]	[ ]	Human research participants
[x]	[ ]	Clinical data
[x]	[ ]	Dual use research of concern

Methods

n/a	Involved in the study	
[x]	[ ]	ChIP-seq
[x]	[ ]	Flow cytometry
[x]	[ ]	MRI-based neuroimaging

Antibodies

Antibodies used	Antibodies used for Western blotting:<br>Protein name/source/Catalog number/dilution/host species<br>α-Synuclein/BD Bioscience/clone 42/610787/1:1000/Mouse, Tyrosine hydroxylase/Pel-Freeze Biologicals/P40101/1:2000/Rabbit, Inducible nitric oxide synthase (iNOS)/BD Bioscience/clone 6/610329/1:1000/Mouse, Ionized calcium binding adaptor molecule 1 (IBA1)/Abcam/ab5076/1:1000/Goat, Glial fibrillary acidic protein (GFAP)/Santacruz/Sc-6171/1:1000/Goat, Interleukin-1β/Santacruz/Sc-7884/1:1000/Goat, Toll like receptor 2 (TLR2) /Abcam/ab16894/T2.5/1:1000/Mouse, MyD88/Santacruz/clone E-11/sc-74532/1:1000/Mouse, Actin/Abcam/ab8226/1:10000/Mouse.<br>Antibodies used for immunostaining:<br>α-Synuclein/Abcam/clone MJFR1/ab138501/1:500/Rabbit, Phospho ser129 α-synuclein/Abcam/ab51253/1:5000/Rabbit, Tyrosine hydroxylase/Pel-Freeze Biologicals/P40101/1:1000/Rabbit, Inducible nitric oxide synthase (iNOS)/BD Bioscience/610329/1:200/Mouse, Ionized calcium binding adaptor molecule 1/Abcam/ab5076/1:1000/Goat, Glial fibrillary acidic protein/Abcam/ab53554/1:1000/Goat, Toll like receptor 2/Abcam/ab16894/1:200/Mouse, Acetylated p65/Abcam/ab19870/1:500/Rabbit.<br>Antibodies used for ChIP assay:<br>p65/Santa Cruz Biotechnology/Clone F-6/sc-8008/2 ug per reaction, p50/Santa Cruz Biotechnology/clone E-10/sc-8414/2 ug per reaction, p300/Santa Cruz Biotechnology/clone F-4/sc-48343/2 ug per reaction, CBP/Santa Cruz Biotechnology/clone G-8/sc-365387/2 ug per reaction, RNA polymerase II/Santa Cruz Biotechnology/clone 8WG16/sc-56767/2 ug per reaction.
Validation	All primary antibodies were commercially available and referenced by multiple studies. According to BD Bioscience, anti-α-Synuclein

antibody (cat# 610787) has been cited in 5 publications. The other anti-α-Synuclein antibody (clone MJFR1) from Abcam has been cited in 6 articles. Phospho ser129 α-synuclein antibody from Abcam has been cited by 173 publications. Similarly, anti-tyrosine hydroxylase antibody from Pel-Freeze Biologicals has been cited in 17 publications. Anti-IBA1 antibody from Abcam has been cited in 668 publications. Anti-GFAP antibody from Abcam has been cited in 185 publications. Anti-TLR2 antibody from Abcam has been cited in 21 publications. Anti-MyD88 antibody (Santa Cruz) has been cited in 72 publications. Anti-iNOS antibody from BD Bioscience always exhibits 130 KD band in Western blot. Anti-Actin antibody from Abcam has been cited by 1895 publications. Anti-acetylated p65 antibody from Abcam is cited by 75 publications. Anti-p65 antibody from Santa Cruz Biotechnology is cited by 1766 publications. Anti-p50 antibody from Santa Cruz Biotechnology is cited by 306 publications. Anti-p300 antibody from Santa Cruz Biotechnology is cited by 60 publications. Anti-CBP antibody from Santa Cruz Biotechnology is cited by 4 publications. Anti-RNA Polymerase II antibody from Santa Cruz Biotechnology is cited by 69 publications.

Eukaryotic cell lines

Policy information about cell lines

Cell line source(s)	BV-2 cells were obtained from Dr. V. Bocchini, University of Perugia, Italy. Dr. Bocchini and colleagues reported the generation of BV-2 cells in 1992. SH-SY5Y cells were purchased from ATCC. MN9D cells were bought from Sigma.
Authentication	Each cell line was validated by evaluating expression of specific marker proteins using immunostaining and PCR.
Mycoplasma contamination	Mycoplasma contamination was not found in the cell lines.
Commonly misidentified lines (See ICLAC register)	No commonly misidentified lines were used in the study.

Animals and other organisms

Policy information about studies involving animals; ARRIVE guidelines recommended for reporting animal research

Laboratory animals	WT (C57BL6), TLR2 KO (B6.129-Tlr2tm1Kir/J) and A53T α-syn transgenic line M83 (B6;C3-Tg(Prnp-SNCA*A53T)83Vle/J) were used from both sexes in the study. Mice were maintained at room temperatures of 65-75°F (~18-23°C) with 40-60% humidity. Mice were kept on a 14/10 h light/dark cycle and given a continuous supply of food and water.
Wild animals	No wild animals were used in the study.
Field-collected samples	No field collected samples were used in the study.
Ethics oversight	Animal maintenance, breeding, surgery procedures were performed in accordance with the National Institutes of Health guidelines and were approved by the Institutional Animal Care and Use committee of the Rush University Medical Center.

Note that full information on the approval of the study protocol must also be provided in the manuscript.
