# Supplementary material for: Selective targeting of the TLR2/MyD88/NF-κB pathway reduces α-synuclein spreading in vitro and in vivo
Source: Nat Commun. 2021 Sep 10;12:5382. doi: 10.1038/s41467-021-25767-1 (PMC8433339; doi:10.1038/s41467-021-25767-1)

Fig 1b

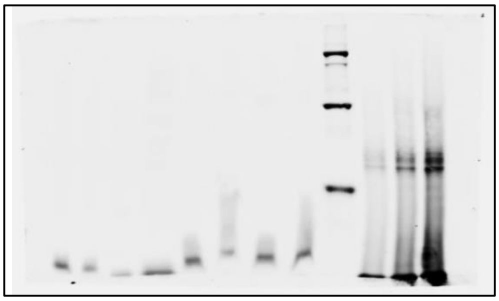

Fig 1c

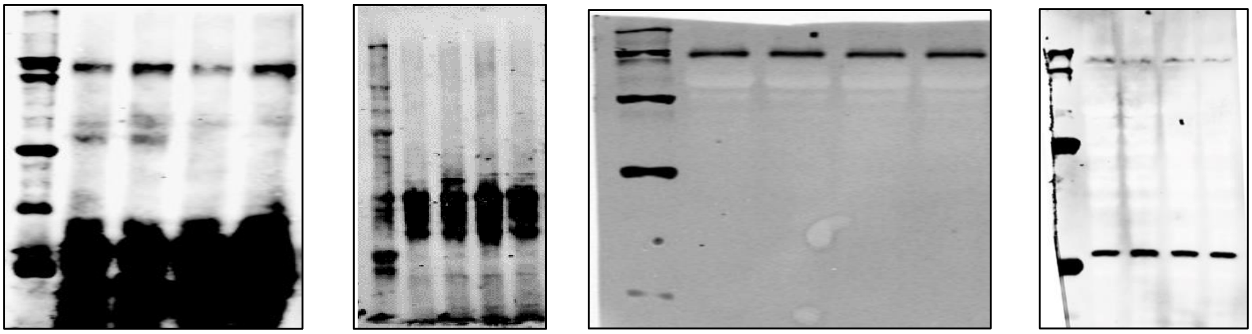

IP: MyD88
IB: TLR2

IP: IgG
IB: TLR2

Input
IB: TLR2

Input
IB: MyD88

Fig 1e

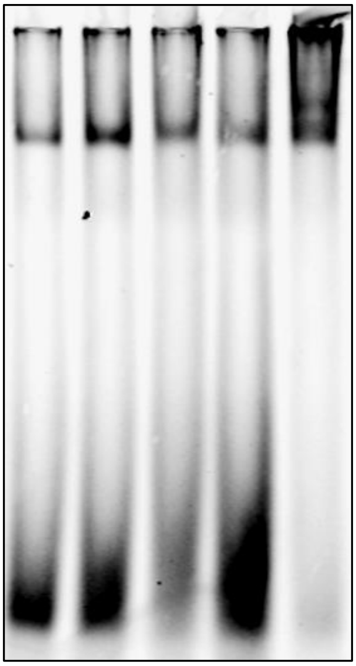

Fig 2a
Syn sol

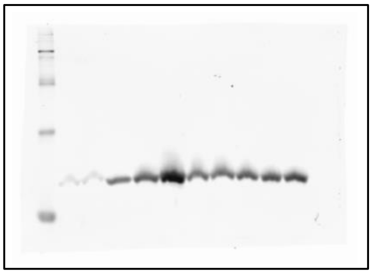

Fig 2a
Actin sol

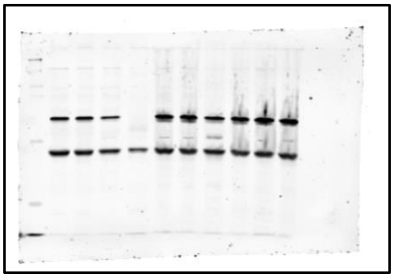

Fig 2b

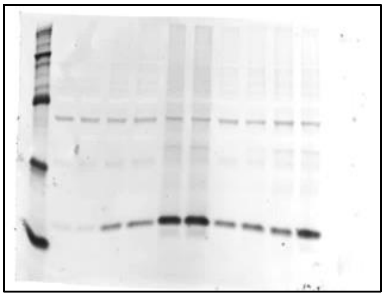

Fig 2b
Actin Insol

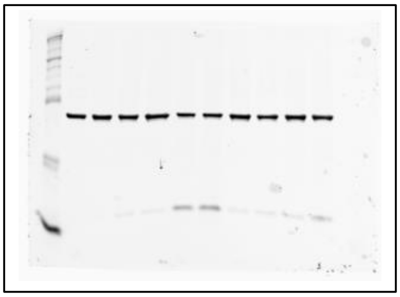

Fig 2i
Syn sol

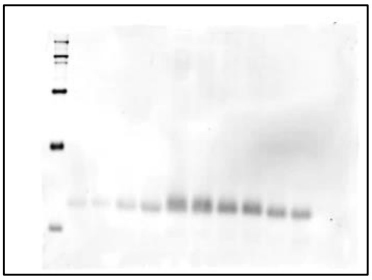

Fig 2i
Actin sol

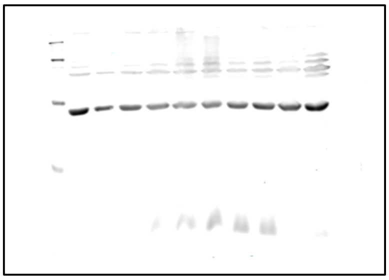

Fig 2j
Syn Insol

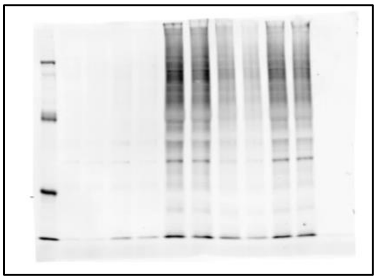

Fig 2j
Actin Insol

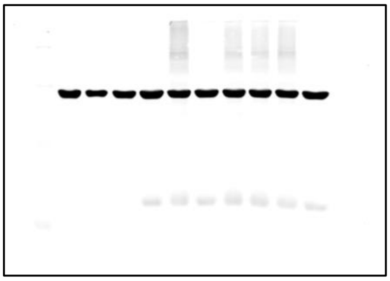

Fig 3B
Syn sol

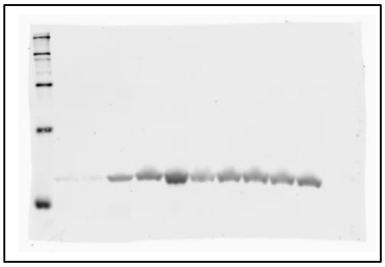

Fig 3B
Actin sol

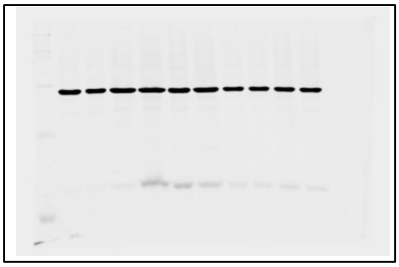

Fig 3C
Syn Insol
High exposure

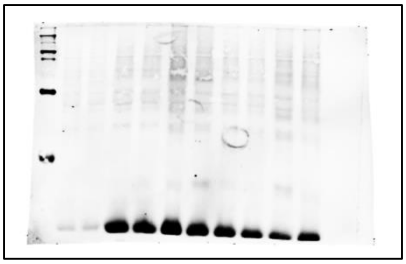

Fig 3C
Syn Insol
Low exposure

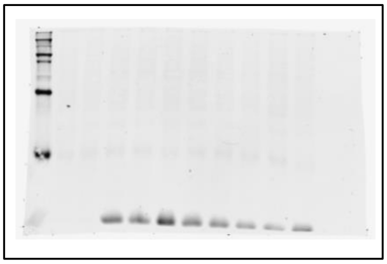

Fig 3C
Actin

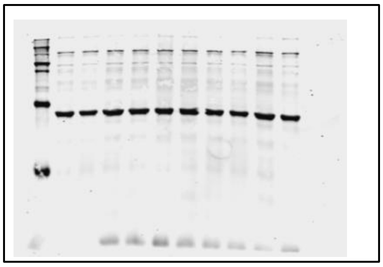

Fig 4C
TH

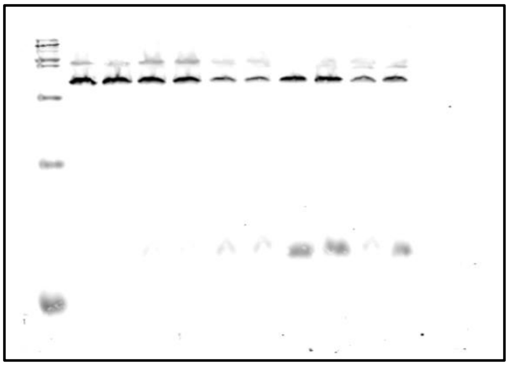

Fig 4C
Actin

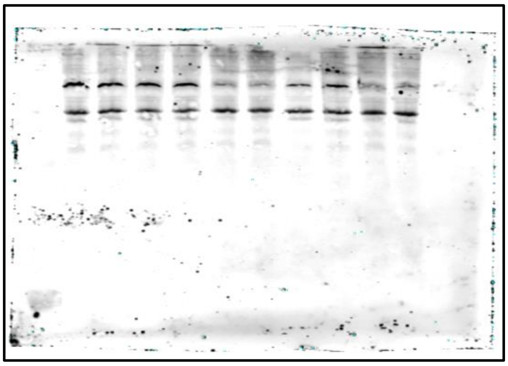

Fig 4G
TH

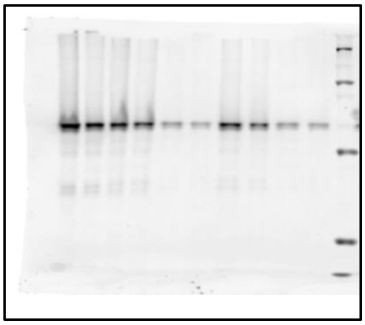

Fig 4G
Actin

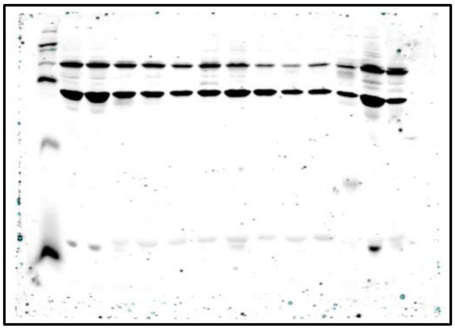

Fig 5a α-Syn

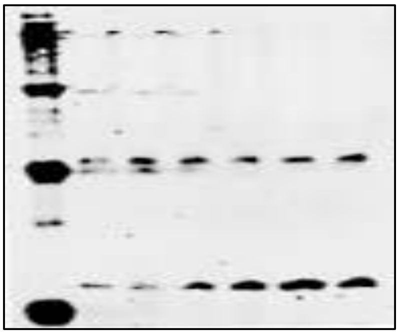

Fig 5a Actin

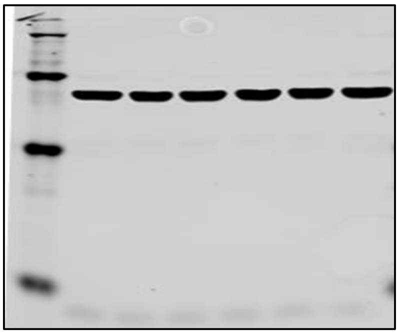

Fig 5i

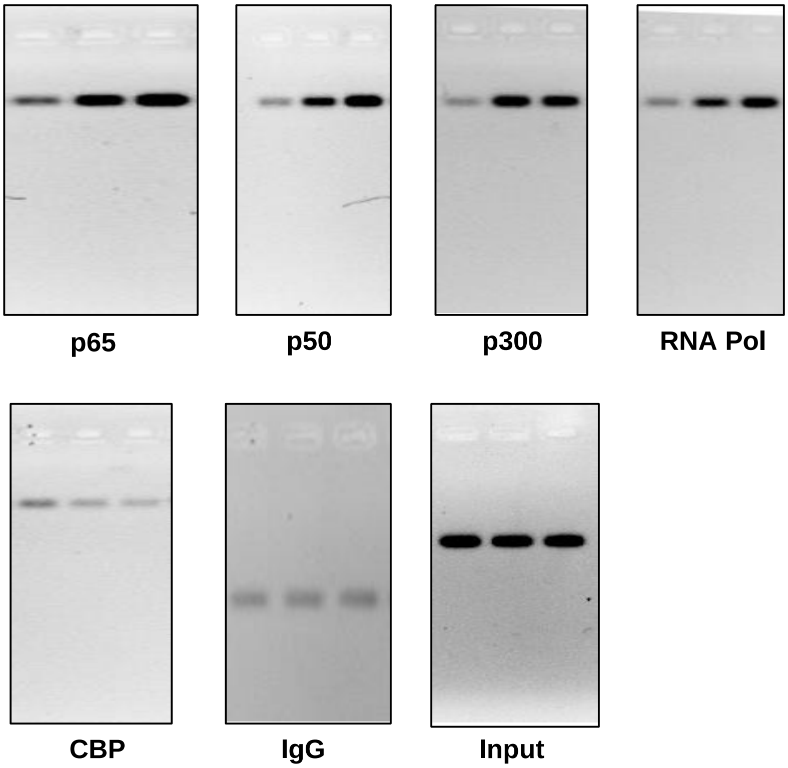

Fig 6h

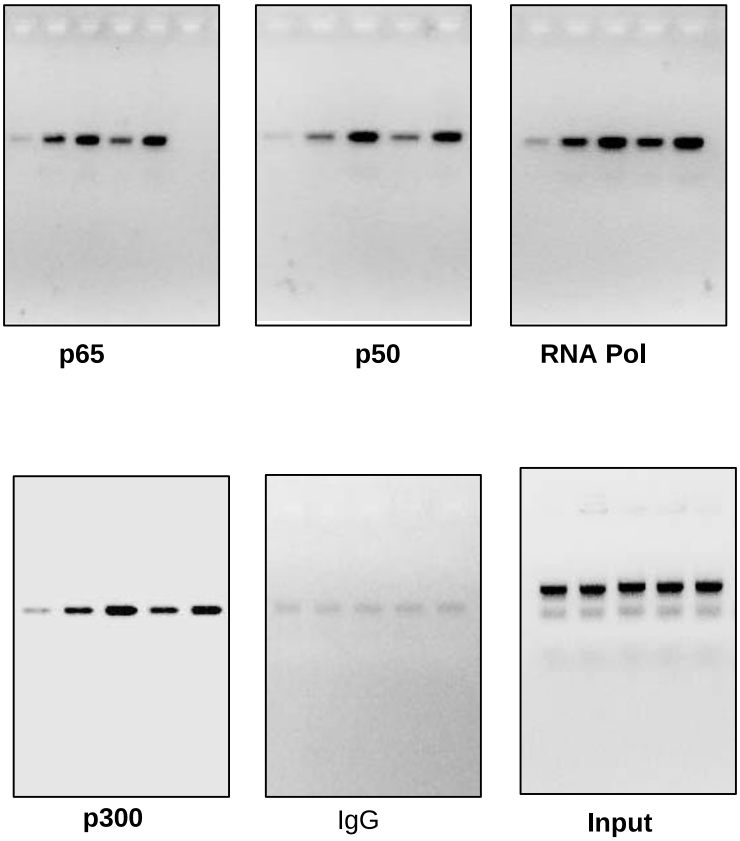

Fig 7C
Syn sol

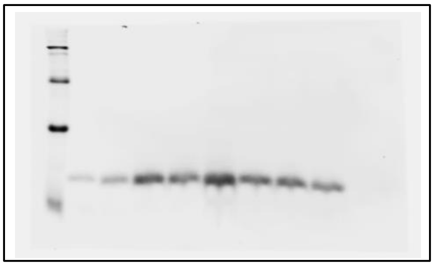

Fig 7C
Actin sol

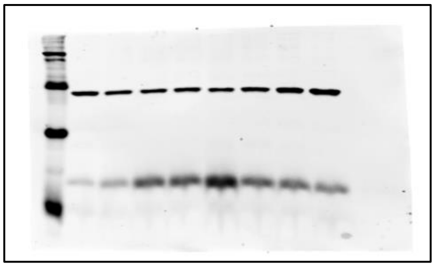

Fig 7D
Syn Insol

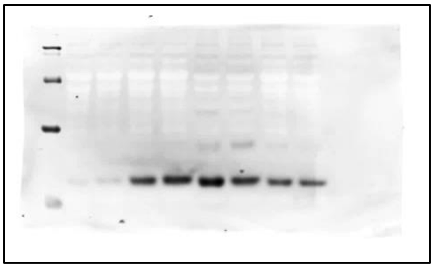

Fig 7D
Actin Insol

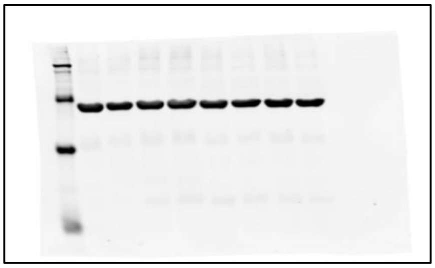

Fig S5F

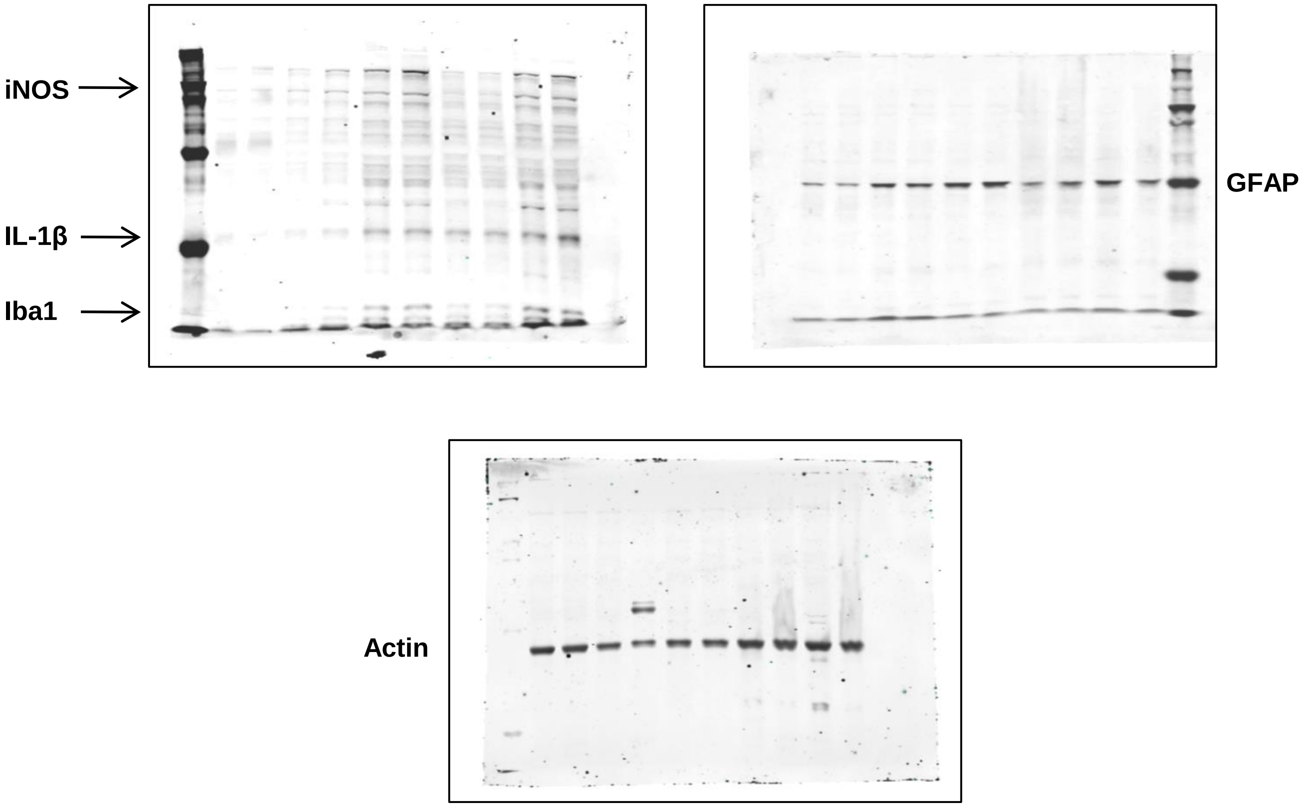

Fig S10A Syn sol

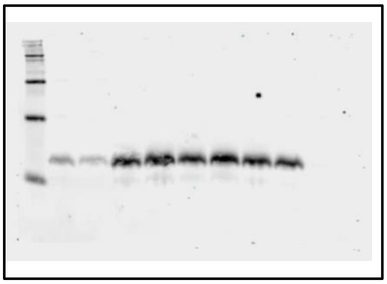

Fig S10A Actin sol

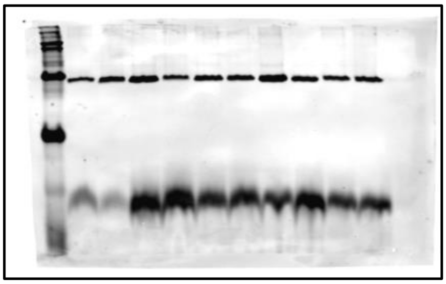

Fig S10C Syn insol
High exposure

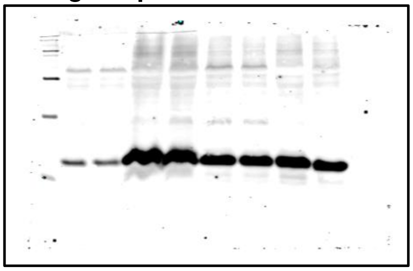

Fig S10C Syn insol
low exposure

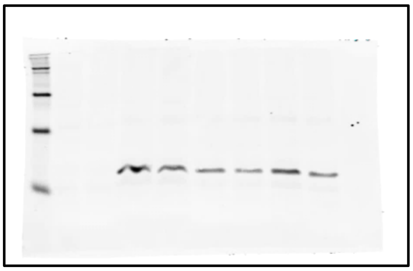

Fig S10C Actin insol

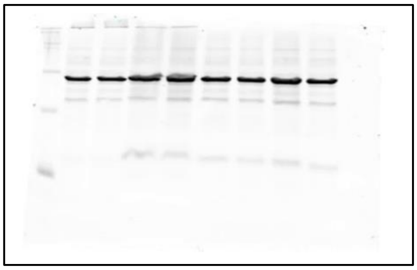

Fig S10K iNOS, IL-1β

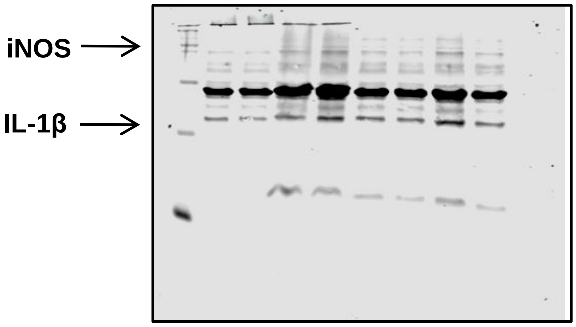

Fig S10K Actin

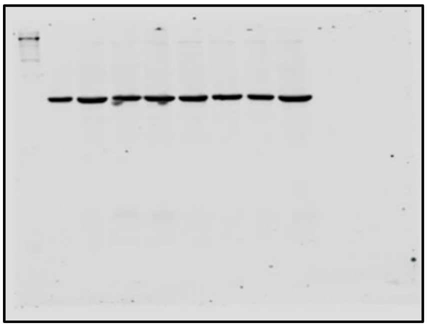

Fig S11A Syn sol

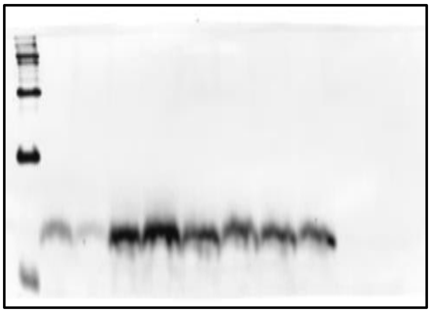

Fig S11A Actin

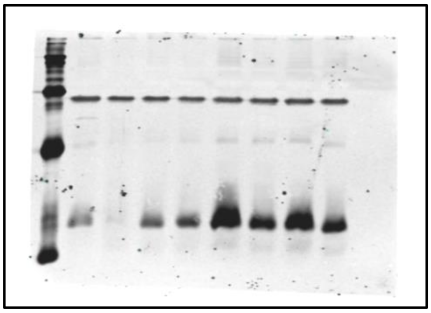

Fig S11C Syn insol

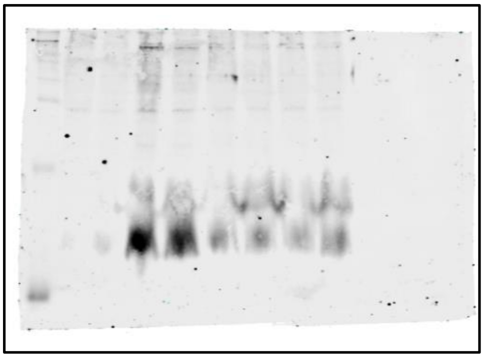

Fig S11C Actin insol

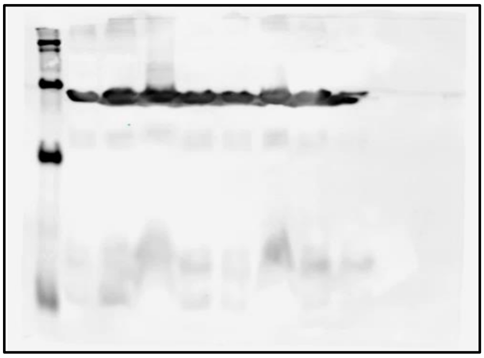

Fig S11K iNOS

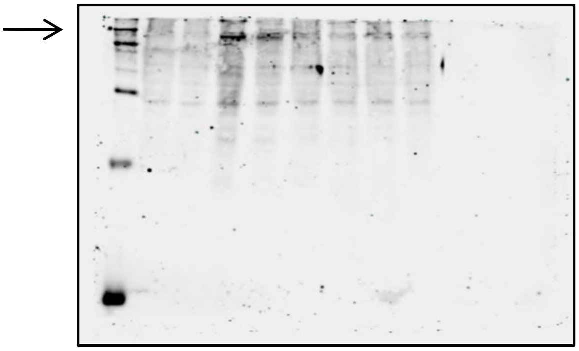

Fig S11K IL-1β

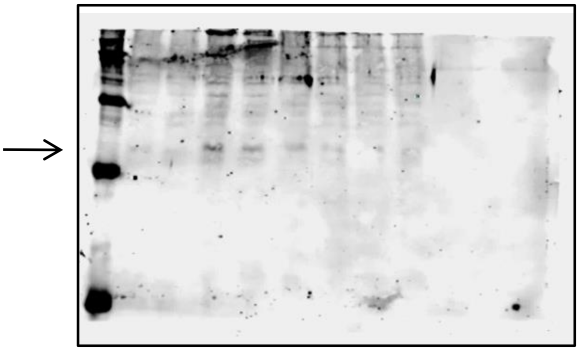

Fig S11K Actin

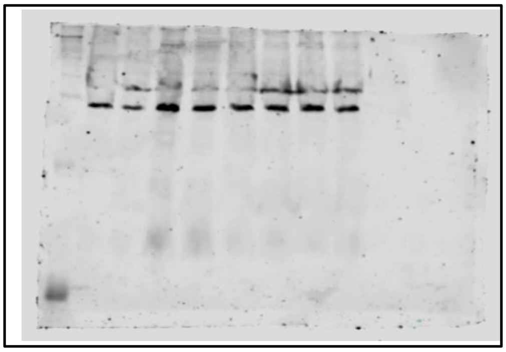

Fig. S12A α-Syn

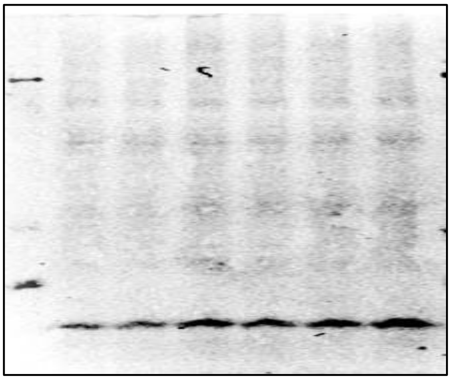

Fig. S12A Actin

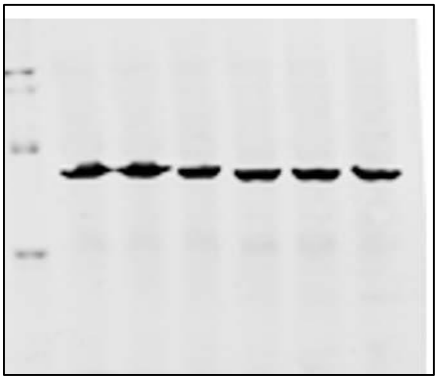

Fig S13D α-Syn

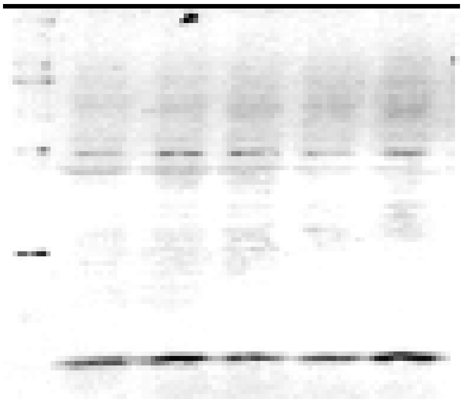

Fig S13D Actin

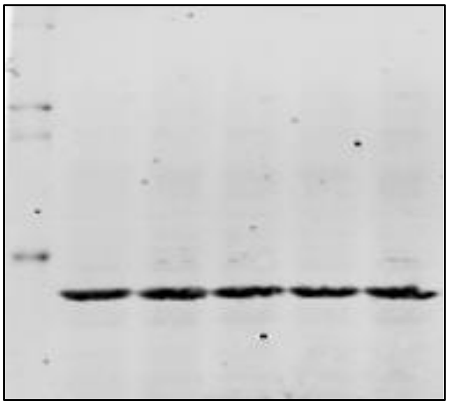

Fig S13f

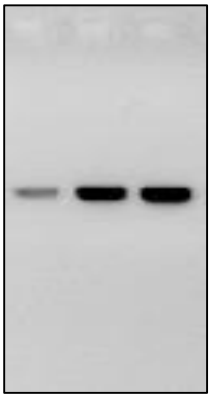

p65

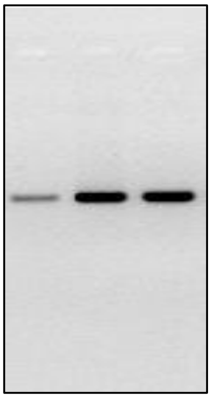

p50

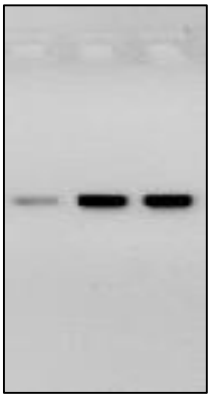

p300

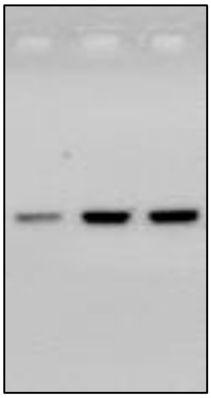

RNA Pol

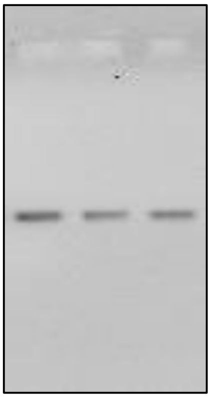

CBP

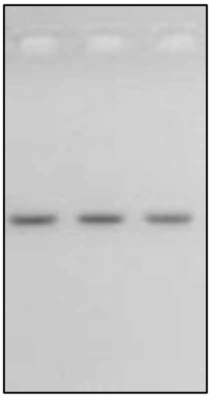

IgG

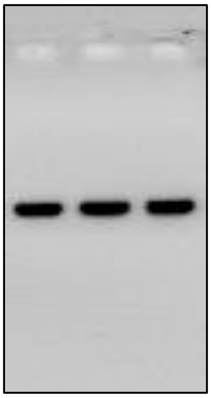

Input

Fig S14a

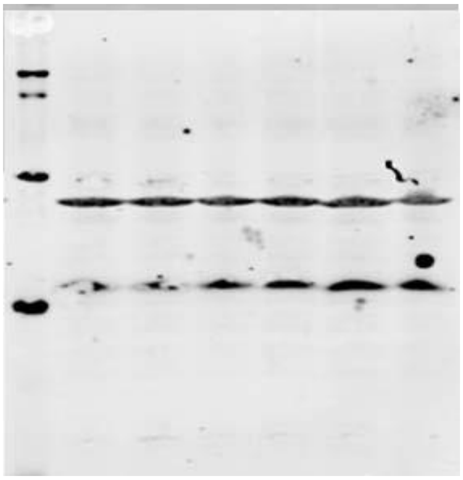

Fig S18B TH

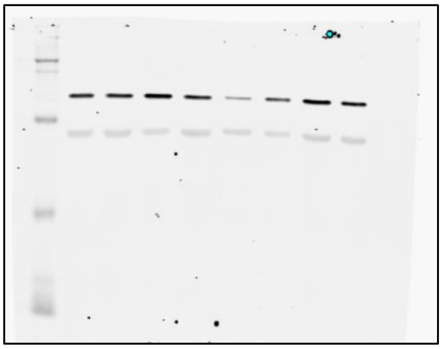

Fig S18B Actin

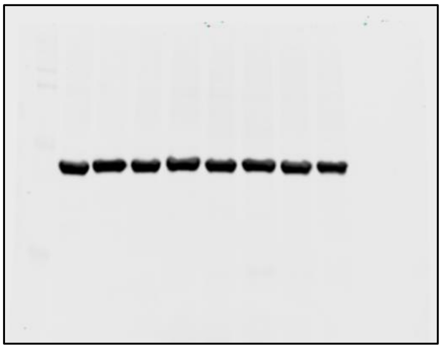

Supplement: Supplementary file 4 — Source Data [file 41467_2021_25767_MOESM4_ESM.zip › Full blots.pdf]
